# The intellectual disability gene PQBP1 rescues Alzheimer’s disease pathology

**DOI:** 10.1038/s41380-018-0253-8

**Published:** 2018-10-03

**Authors:** Hikari Tanaka, Kanoh Kondo, Xigui Chen, Hidenori Homma, Kazuhiko Tagawa, Aurelian Kerever, Shigeki Aoki, Takashi Saito, Takaomi Saido, Shin-ichi Muramatsu, Kyota Fujita, Hitoshi Okazawa

**Affiliations:** 1 Department of Neuropathology, Medical Research Institute, Tokyo Medical and Dental University, 1-5-45 Yushima, Bunkyo-ku, Tokyo, 113-8510 Japan; 2 Department of Radiology, Juntendo University School of Medicine, 2-1-1 Hongo, Bunkyo-ku, Tokyo, 113-8421 Japan; 3 Laboratory for Proteolytic Neuroscience, Center for Brain Science, RIKEN, 2-1 Hirosawa, Wako, Saitama, 351-0198 Japan; 4 Department of Neurology, Jichi Medical University, 3311-1 Yakushiji, Shimotsuke, Tochigi, 329-0496 Japan; 5 Center for Brain Integration Research, Tokyo Medical and Dental University, 1-5-45 Yushima, Bunkyo-ku, Tokyo, 113-8510 Japan

**Keywords:** Neuroscience, Molecular biology

## Abstract

Early-phase pathologies of Alzheimer’s disease (AD) are attracting much attention after clinical trials of drugs designed to remove beta-amyloid (Aβ) aggregates failed to recover memory and cognitive function in symptomatic AD patients. Here, we show that phosphorylation of serine/arginine repetitive matrix 2 (SRRM2) at Ser1068, which is observed in the brains of early phase AD mouse models and postmortem end-stage AD patients, prevents its nuclear translocation by inhibiting interaction with T-complex protein subunit α. SRRM2 deficiency in neurons destabilized polyglutamine binding protein 1 (PQBP1), a causative gene for intellectual disability (ID), greatly affecting the splicing patterns of synapse-related genes, as demonstrated in a newly generated PQBP1-conditional knockout model. PQBP1 and SRRM2 were downregulated in cortical neurons of human AD patients and mouse AD models, and the AAV-PQBP1 vector recovered RNA splicing, the synapse phenotype, and the cognitive decline in the two mouse models. Finally, the kinases responsible for the phosphorylation of SRRM2 at Ser1068 were identified as ERK1/2 (MAPK3/1). These results collectively reveal a new aspect of AD pathology in which a phosphorylation signal affecting RNA splicing and synapse integrity precedes the formation of extracellular Aβ aggregates and may progress in parallel with tau phosphorylation.

## Introduction

Alzheimer’s disease (AD) is pathologically defined by extracellular beta amyloid (Aβ) aggregates, which is called “senile plaque” in human pathology. Therefore, therapeutic drugs are being developed with the aim of removing extracellular Aβ aggregates. However, multiple Phase III clinical trials have failed to recover memory and cognitive function in AD patients using trial drugs, such as anti-Aβ antibody, despite their efficacy in decreasing Aβ aggregates, as determined by amyloid positron emission CT (PET). This discrepancy demands an alternative explanation for the time sequence of Aβ aggregate formation and irreversible neuronal damage, and it highlights the significance of preclinical or prodromal stages of AD.

In previous work, we unexpectedly found that the phosphorylation status of certain proteins changes before the formation of Aβ aggregates in the extracellular space [1]. Two of the three proteins identified are MARCKS and the homolog MARCKS-like (MarcksL). The third protein is serine/arginine repetitive matrix 2 (SRRM2) also known as SRm300, which was identified as a scaffold protein for multiple splicing factors including snRNPs and SR family proteins [2]. SR proteins possess RNA binding motifs that mediate interaction with specific sequences of target pre-mRNAs [3], and snRNPs form a splicing complex for the cleavage of the pre-mRNA [2]. Therefore, SRRM2 may be a splicing regulator, although its exact function remains largely unknown.

Polyglutamine binding protein 1 (PQBP1), a polyglutamine tract-binding protein [4, 5], is involved in the pathology of polyglutamine diseases, such as spinocerebellar ataxia type-1^6^ and Huntington’s disease [5], and mutations in PQBP1 cause syndromic intellectual disability (ID) with microcephaly named Renpenning’s syndrome and several other syndromes or non-syndromic IDs [7–10].

PQBP1 interacts with RNA polymerase II [6]. In addition, it is involved in the B-complex and in RNA splicing [11, 12], and it interacts with several splicing factors [13–15]. Conditional knock-out of *Pqbp1* in neural stem/progenitor cells in mice reproduces microcephaly and ID. Cell cycle elongation, caused by splicing-based impairment of cell cycle-related genes, underlies the microcephaly [16]. PQBP1 deficiency also impairs cilia formation via GTPase dynamin 2 in postmitotic neurons [17], and causes dendrite shortening [15]. PQBP1 was recently identified as an intracellular receptor for the cDNA of the HIV virus, which induces immune responses in dendritic cells [18]. In addition, it is suggested to be involved in the HEXIM1-DNA-PK-paraspeckle components-ribonucleoprotein (HDP-RNP) complex [19].

Here, we showed that SRRM2 phosphorylation at Ser1068 increases at the early stage of AD pathology associated with the translocation of SRRM2 to the cytoplasm, destabilizes the spliceosome component PQBP1, broadly affects RNA splicing of synapse genes, and eventually leads to cognitive impairment. Concurrent deficiency of SRRM2 and PQBP1 was confirmed in human AD brains. Restoration of PQBP1 by an adeno-associated virus (AAV) vector recovered synaptic structures and cognitive function in AD model mice.

## Materials and Methods

### Brain samples from the AD mouse model and AD patients

Transgenic 5xFAD mice carrying mutant human APP770 with Swedish (KM670/671NL), Florida (I716V), and London (V717I) triple mutations, as well as human PS1 harboring double mutants (M146L and L285V) under the mouse Thy1 promoter, and their background mice (B6/SJL) were used [20].

Generation of APP-KI mice (*App*^*NL-G-F/NL-G-F*^ mice) was described previously [21]. Briefly, *App*^*NL-G-F/NL-G-F*^ mice carry the Arctic, Swedish, and Beyreuther/Iberian mutations of the mouse *App* gene [Swedish mutation (KM670/671NL) in exon 16, and Beyreuther/Iberian (I716F) and Arctic (E693G) mutations in exon 17]. *App*^*NL-G-F/NL-G-F*^ mice also carry the humanized Aβ sequence (G676R, F681Y, and R684H), which is located between the β-cleavage site and the γ-cleavage site (672–713 a.a.) of the *App* gene. C57BL/6 mice were the background strain.

For AD patient samples, informed consent and ethics committee approval were obtained to examine autopsy specimens from AD patients and control patients without neurological disorders. A neuropathologist established the pathological diagnosis of the brains based on immunohistochemistry. AD brains lacking other pathological changes such as Lewy bodies or TDP43 aggregates were selected for use.

### Generation of Pqbp1-cKO mice

Pqbp1 gene conditional knock-out mice were generated as previously described [16]. Briefly, a target vector was generated by PCR from a BAC library (ID:RP23-404N15) and constructed using a 3.6-kb 5′ fragment containing exons 1 and 2, a neomycin resistance cassette flanked by Flp recognition target sites, a 3.9-kb fragment containing exons 3–7 between two LoxP sites, a 4.1-kb non-coding 3′ fragment, and a diphtheria toxin A gene. The target vector was introduced into ES cells (C57BL/6) using electroporation, selected with G418, and cloned. Genomic DNAs of cloned ES cells were analyzed by PCR and Southern blotting. The selected ES cells were injected into C57BL/6 blastocysts, and chimeric mice were cross-bred with C57BL/6 mice. Pqbp1-floxed mice were generated by removing the neomycin resistance cassette by cross-breeding with CAG-FLPe recombinase transgenic mice [22]. Pqbp1 conditional knockout mice were generated to cross-breed the Pqbp1-floxed heterozygous or homozygous female mice with Synapsin1-Cre transgenic heterozygous male mice [B6.Cg-Tg (Syn1-Cre) 671Jxm/J; The Jackson Laboratory, Bar Harbor, ME, USA]. Impairment of Pqbp1-cKO mice in behavioral tests such as the open field test, light-dark box, elevated plus maze test, fear conditioning test, water maze test, and rotarod test was reported in Supplementary Fig. 11 of our previous paper [16].

### Generation of mutated human iPS cell lines of APP KM670/671NL

Human normal iPS cells (ASE-9203, Applied StemCell, Inc., CA, USA) were transfected with a DNA mixture of plasmids expressing gRNA (5′-GGAGATCTCTGAAGTGAAGATGG-3′) and the cas9 gene along with single-stranded oligodeoxynucleotides (for human APP KM670/671NL, 5′-TGGTTGTCCTGCATACTTTAATTATGATGTAATACAGGTTCTGGGTTGACAAATATCAAGACGGAGGAGATCTCTGAAGTGAATCTGGATGCAGAATTCCGACATGACTCAGGATATGAAGTTCATCATCAAAAATTGGTACGTAAAATAATTTACCTCTTTC-3′) for donor DNA. The cas9 gene was fused to the 2A peptide and GFP gene. Cells were electroporated using a Neon system (Thermo Fisher Scientific Inc., MA, USA) with the conditions 1200 V/30 ms/1pulse. Cells were selected with 0.4 μg/mL Puromycin for 24–48 h post transfection and subjected to a colony cloning process by picking and seeding each visible GFP positive colony into a well of a 96-well plate. The cells were allowed to grow for 7–10 days, or until a workable sized colony was formed. A portion of cells from each colony was subjected to genotype analysis. Briefly, genomic DNA from single cell colonies was isolated and used to amplify a 308 bp DNA fragment using the primers 5′-GCATGTATTTAAAGGCAGCAGAAGC-3′ and 5′-CAATGCTTGCCTATAGGATTACCATGAAAACATG-3′. PCR fragments were subjected to Sanger sequencing. Positive clones were expanded, and a portion of the cells was resubmitted for sequencing to confirm the desired genotype.

### Primary neuron culture

Mouse primary cerebral neurons were prepared from embryonic day 15 C57BL/6J mice embryos. 4 to 6 total cerebral cortexes were dissected, incubated with 0.05% trypsin in 4 mL of PBS (Thermo Fisher Scientific Inc., #25200056, MA, USA) at 37 °C for 15 min, dissociated by pipetting. The cells were passed through a 70 μm cell strainer (Thermo Fisher Scientific Inc., #22-363-548, MA, USA), collected by centrifugation, and cultured in neurobasal medium (Thermo Fisher Scientific Inc., MA, USA) containing 2% B27, 0.5 mM L-glutamine, and 1% Penicillin/Streptomycin in the presence of 0.5 μM AraC. For western blot, immunohistochemistry and immunoprecipitation, primary neurons at seven days (DIV 7) were used.

Preparation of adult mouse neurons was performed according to the method reported previously [23]. In brief, cerebral cortexes were dissected from 5xFAD mice at 1 month of age, minced to 1 mm cubes, incubated with 2 mg/mL papain (Sigma-Aldrich, P4762, St. Louis, MO, USA) and 0.5 mM L-glutamine in hibernate A medium (Thermo Fisher Scientific Inc., MA, USA) at 30 °C for 30 min, dissociated by pipetting with pasteur pipette, passed through 70 μm then 40 μm cell strainer (Thermo Fisher Scientific Inc., MA, USA). Dissociated cells were directly applied to FACS analysis to separate neurons, glia and other cells, and used for western blot. Separated neurons were also fractionated to nucleus and cytoplasm as described in the following.

### Fluorescence activated cell sorting

Cells prepared from the cerebral cortex of 5xFAD mice at 1 month of age were stained with mouse anti-GFAP (1:500, Sigma-Aldrich, S9205) and rabbit anti-MAP2 (1:250, Abcam, ab32454, Cambridge, UK) followed by secondary antibodies (anti-mouse IgG Alexa488 (1:500, Thermo Fisher Scientific Inc, #A212C2) and anti-rabbit IgG Alexa647 (1:500, Thermo Fisher Scientific Inc. #A31573), respectively. Stained cells were sorted to neurons, glia and other cells by MoFlo XDP (Beckman Coulter, CA, USA). The neurons proceeded further to subcellular fractionation as described below. Cytosolic and nuclear fractions were diluted with sample buffer and boiled for 5 min at 95 °C, then samples were subjected to SDS-PAGE.

### Subcellular fractionation

Neurons were washed with ice-cold PBS, and lysed with hypotonic buffer (20 mM HEPES pH 7.9, 10 mM KCl, 2 mM MgCl_2_, 0.3% NP-40, 1 mM DTT, 1 mM EDTA, 0.4% protease inhibitor cocktail, and 1 mM PMSF). After 20 min of incubation on ice, lysates were centrifuged at 15,000×*g* for 20 min at 4 °C. The supernatants were stored as the cytoplasmic fraction. The pellets were resuspended with nuclear extract buffer (20 mM HEPES pH 7.9, 420 mM NaCl, 1 mM EDTA, 2 mM MgCl_2_, 10% glycerol, 1 mM DTT, and 0.4% protease inhibitor cocktail). After 20 min of incubation on ice, lysates were centrifuged at 15,000×*g* for 20 min at 4 °C. The supernatants were stored as the nuclear fraction. Cytoplasmic and nuclear extracts were diluted with sample buffer, and boiled for 5 min at 95 °C.

### Plasmid and virus vector construction

To construct mouse pEGFP-C1-SRRM2-WT, mouse SRRM2-pFLSI (DNAFORM, AK090123, Kanagawa, Japan) was amplified using the primers 5′-atgcgaattctatgttgctggaaaaggatg-3′ and 5′-gctcctccaggtctccataaggatccgcat-3′. After digestion with *EcoR*I and *Kpn*I, SRRM2-pFLSI was subcloned into pEGFP-C1 (Clontech, Mountain View, CA, USA). Mutagenesis was carried out with pEGFP-C1-SRRM2-WT. The primer sets for S1068A, S1068D, or S1068E mutagenesis were as follows:

(S1068A) forward: 5′-tcttcagctccagtcactgagctgaca-3′, reverse: 5′-agcctttccagatcttcagctccagtc-3′;

(S1068D) forward: 5′-agatcttcagatccagtcactgagctgaca-3′, reverse: 5′-ggtagcctttccagatcttcagatcca-3′;

(S1068E) forward: 5′-tccagatcttcagaaccagtcactgag-3’, reverse: 5′-tggatctgaagatctggaaaggctacc-3′.

The AAV-hSYN1-GFP vector was a kind gift from Prof. Muramatsu (Jichi medical university). The AAV-hSYN1-rVAMP2-mCherry vector was purchased from Vector BioLabs (Malvern, PA, USA).

### Mass spectrometry

Using 5xFAD and littermate control (non-transgenic) mice, a comprehensive proteomics analysis was performed with brain tissues of male mice at 1, 3, 6, and 12 months of age as previously described [1]. Briefly, brain extracts were denatured by detergent and heat treatment, reduced to block cysteine bonds, and digested with trypsin. The phosphopeptides were enriched using the Titansphere Phos-TiO kit (GL Sciences Inc., Japan) and labeled using an iTRAQ Reagent multiplex kit (SCIEX Ins.). Liquid chromatography separated the enriched and labeled phosphopeptides using a strong cation exchange (SCX) column. Each fraction was separated using a DiNa Nano-Flow LC system (KYA Technologies Corporation, Japan) and 0.1 × 100 mm C18 columns (KYA Technologies Corporation). The ion spray voltage applied to the sample from the Nano-LC to the Triple TOF 5600 System (SCIEX Ins.) was 2.3 kV. The information-dependent acquisition setting was 400–1250 *m*/*z*.

### Anti-phosphorylated SRRM2 antibody

Two phospho-peptides were synthesized: C-KGSLSRSSpSPVTE (phospho Ser 1068 SRRM2) and C-QPEVALKRVPpSPT (phospho Ser 2535 SRRM2), in which a Cys residue was added at the N-terminus and conjugated to KLH. Two New Zealand White rabbits (SPF) per antigen were administered 1 mg/ml antigen subcutaneously in three to five sites per animal on the back. Injections were given as emulsions in Complete Freund’s Adjuvant (CFA) with 350 µg phospho-peptides on days 0 and 7 or Incomplete Freund’s Adjuvant (IFA) with 150 µg phospho-peptides on days 14 and 21. Rabbits were boosted with 150 µg antigens in 0.9% NaCl on days 28, 35, and 42. Pre-immune serum was collected on day 0 and small-scale serum on days 28 and 42, and they were tested by ELISA using antigen phospho-peptides. Large-scale sera were collected on day 49. Sera were applied to a non-phosphopeptide-conjugated resin and the flow-through fractions were applied to a phosphopeptide-conjugated resin. Anti-phospho Ser 1068 and 2535 SRRM2 antibodies were purified by specific phospho and non-phospho peptides.

### Immunohistochemistry

Mouse brains were fixed in 4% paraformaldehyde for 12 h. Paraffin sections (thickness, 5 μm) were de-paraffinized in xylene, re-hydrated, dipped in 10 mM citrate buffer (pH 6.0), and microwaved at 120 °C for 15 min. The sections were incubated sequentially with 0.5% TritonX-100 in PBS for 30 min at room temperature (RT) for membrane permeation. After blocking with 10% FBS containing PBS, sections were incubated with primary antibody overnight, and finally with secondary antibodies for 60 min at RT. The antibodies used for immunohistochemistry were diluted as follows: rabbit anti-phospho-SRRM2 [1:150, GL Biochem (Shanghai) Ltd., Shanghai, China]; rabbit anti-PQBP1 (1:100, Bethyl Laboratories, A302-801A, Montgomery, TX, USA); mouse anti-beta Amyloid 6E10 (1:2000, BioLegend, SIG-39320-200, San Diego, CA, USA); anti-beta Amyloid 82E1 (1:1000, IBL, 10323, Fujioka, Gunma, Japan); Cy3-conjugated anti-mouse IgG (1:500, Jackson Laboratory, 715-165-150, Bar Harbor, ME, USA); Alexa Fluor 488-conjugated anti-rabbit IgG (1:1000, Molecular Probes, A21206, Eugene, OR, USA).

### Immunocytochemistry and hyperosmolar treatment

HeLa cells or mouse primary cortical neurons (E15) were washed with PBS before fixation for 2 min at RT with cytoskeletal buffer (10 mM PIPES pH 6.8, 300 mM sucrose, 100 mM NaCl, 3 mM MgCl_2_, 1 mM EGTA, and protease inhibitor cocktail) containing 0.5% TritonX-100 [24]. For sorbitol treatment, 0.4 M sorbitol was applied to HeLa cells or primary neurons for 60 min before fixation. Then, cells were fixed in cytoskeletal buffer containing 0.4% formaldehyde for 20 min at RT, washed three times in PBS, blocked with blocking buffer (50 mM Tris-HCl pH 6.8, 150 mM NaCl, and 0.1% TritonX-100) containing 5 mg/mL BSA for 60 min at RT, and incubated with primary antibody for 60 min at RT, and finally with secondary antibodies for 60 min at RT. The antibodies used for immunocytochemistry were diluted as follows: rabbit anti-phospho-SRRM2 (Ser1068, 1:500); rabbit anti-PQBP1 (1:150, Bethyl, A302-801A); rabbit anti-SRm300 (1:150, Santa Cruz Biotechnology, sc-292291, Dallas, TX, USA); rabbit anti-SRRM2 (1:300, Abcam, ab122719, Cambridge, UK); mouse anti-SC35 (1:1000, Sigma-Aldrich, S4045, St. Louis, MO, USA); Cy3-conjugated anti-mouse IgG (1:500, Jackson Laboratory, 715-165-150); Alexa Fluor 633-conjugated anti-rabbit IgG (1:1000, Molecular Probes, A21070); Alexa Fluor 488-conjugated anti-rabbit IgG (1:1000, Molecular Probes, A11008); Zenon Alexa Fluor 488 Rabbit IgG Labeling Kit (Thermo Fisher Scientific, Z25302, Waltham, MA, USA).

### Western blot analysis

Mouse brain extracts were obtained at the indicated ages using extraction buffer containing 2% SDS, 1 mM DTT, and 10 mM Tris-HCl (pH7.5), and homogenized using 20 strokes of a Dounce glass homogenizer on ice, as described previously [1]. Cell lysates (mouse primary neurons and HeLa cells) were obtained using the same extract buffer used for mice brain extracts, followed by homogenization with a disposable homogenizer (BioMasher II, Nippi, Tokyo, Japan). The crude extracts were centrifuged at 16,000×*g* at 4 °C for 10 min. The protein concentration was measured using the BCA Protein Assay Reagent (Thermo Fish Scientific). For testing ERK1 kinase reactivity in cell extracts, the extraction buffer was exchanged for a kinase reaction buffer [25 mM MOPS (3-morpholinopropane-1-sulfonic acid, pH 7.2), 12.5 mM β-glycerol-phosphate, 25 mM EGTA, 5 mM EDTA, and 0.25 mM DTT] using an Amicon Ultra 3 K filter (Millipore, Billerica, MA, USA). Cell extracts were obtained from 10 cm dishes, and 20% of the volume of extracts was reacted with 25 ng ERK1 (SignalChem, M29-10G, Richmond, BC, Canada) and 500 μM ATP for 2 h at 30 °C. Then, half of reactants were subjected to phosphatase treatment (λPPase, New England BioLabs, P0753S, Ipswich, MA, USA) for another 2 h at 30 °C.

Samples were separated by SDS-PAGE, transferred onto polyvinylidene difluoride membranes (Immobilon-P, Merck Millipore) using the semi-dry method, blocked with 5% milk or 2% BSA in TBST (10 mM Tris/HCl pH 8.0, 150 mM NaCl, and 0.05% Tween-20), and reacted with the following primary and secondary antibodies diluted in Can Get Signal solution (Toyobo, Osaka, Japan). Primary and secondary antibodies were diluted as follows: rabbit anti-phospho-SRRM2 (Ser1068, 1:5000); rabbit anti-SRm300 (1:2000, Santa Cruz Biotechnology, sc-292291); rabbit anti-SRRM2 (1:3000, Abcam, ab122719); mouse anti-PQBP1 (1:500, Santa Cruz Biotechnology, sc-374260); rat anti-TCP1alpha (1:1000, Abcam, ab31911); mouse-anti-SC35 (1:500, Sigma-Aldrich, S4045); rabbit anti-c-Jun (1:1000, Santa Cruz Biotechnology, sc-44); rabbit-anti-HP1alpha (1:3000, Cell Signaling Technology, 2625 S, Danvers, MA, USA); anti-α-tubulin (1:3000, Millipore, T6199); HRP-conjugated anti-mouse IgG (1:3000, GE Healthcare, NA931VA) and anti-rabbit IgG (1:3000, GE Healthcare, NA934VS). ECL prime (GE Healthcare, RPN2232) or SCL select (GE Healthcare, RPN2235) were used to detect the bands using LAS4000 (GE Healthcare).

### Immunoprecipitation

Mouse cortical neurons at 7 days in primary culture were harvested with lysis buffer (10 mM Tris-HCl, 10 mM NaCl, 0.5 mM EDTA, 1% NP-40, and 0.5% protease inhibitor cocktail) and lysates were rotated for 60 min at 4 °C. After 20 min of centrifugation at 12,000 rpm, supernatants were incubated with 1 μg antibody for 12 h at 4 °C. Antibodies were used as follows; rabbit anti-SRm300 (Santa Cruz Biotechnology, sc-292291); mouse anti-PQBP1 (Santa Cruz Biotechnology, sc-374260); mouse-anti-SC35 (Sigma-Aldrich, S4045); rabbit anti-c-Jun (Santa Cruz Biotechnology, sc-44). Protein G Sepharose (GE Healthcare, Buckinghamshire, UK) was incubated for 4 h at 4 °C, and beads were washed with lysis buffer four times. Then, sample buffer was added to the beads, and samples were boiled at 95 °C for 10 min before SDS-PAGE.

### Identification of SRRM2-binding proteins

HeLa cells were transfected with EGFP-empty, EGFP-SRRM2-WT, EGFP-SRRM2-S1068A, EGFP-SRRM2-S1068D, or EGFP-SRRM2-S1068E using Lipofectamine LTX (Thermo Fisher Scientific, #15338100). Forty-eight hours after transfection, cell extracts were obtained using lysis buffer (10 mM Tris-HCl p7.5, 100 mM NaCl, 0.5 mM EDTA, 0.5% NP-40, and 0.5% protease inhibitor cocktail). After 30 min, extracts were centrifuged at 20,000×*g* for 10 min at 4 °C. GFP trap was performed using GFP-Trap_A (ChromoTek, gta-20, Munich, Germany). GFP trap beads were washed with dilute buffer (10 mM Tris-HCl pH 7.5, 100 mM NaCl, 0.5 mM EDTA, and 0.5% protease inhibitor cocktail), and lysates were added to GFP trap beads. After 90 min of rotation at 4 °C, beads were washed with lysis buffer three times, added to an equal volume of sample buffer (0.1 M Tris-HCl pH 7.5, 4% SDS, 20% glycerol, 12% β-mercaptoethanol, and 1% bromophenol blue), and boiled at 95 °C for 10 min.

Co-precipitated proteins with EGFP-SRRM2-WT, EGFP-SRRM2-S1068A, EGFP-SRRM2-S1068D, or EGFP-SRRM2-S1068E were separated by SDS-PAGE using Extra PAGE One Precast Gels (Nacalai Tesque, #13063-74, Kyoto, Japan). Gels were fixed for 15 min at RT using a buffer containing 30% EtOH and 10% acetic acid in MilliQ water. Sliver-staining was performed according to the protocol of Silver Stain for Mass Spectrometry (Thermo Fisher Scientific, #24600). Cropped gels were destained using the Silver Destain Reagent for 30 min, followed by incubation in wash solution (25 mM ammonium bicarbonate and 50% acetonitrile). Gels were incubated with acetonitrile for 10 min, followed by 5 mM TCEP solution for 60 min at 56 °C. Cysteine residues were blocked with 10 mM methyl methanethiosulfonate for 10 min at 25 °C. Then, gels were digested with 13 ng/μl trypsin on ice for 120 min, 100 mM TEAB was added, and tubes with gels were incubated for 12 h at 37 °C. Peptide digestion products were extracted using extraction buffer (5% formic acid in acetonitrile solution), incubated for 15 min at 37 °C, and desalted using a tip column (ZipTip 0.2 μl, U-C18, Millipore). Dried-up samples were re-diluted with 0.1% formic acid and subjected to LC-MS/MS (see “MS spectrometry”).

### Knockdown by siRNA

HeLa cells were treated with SRRM2-siRNA (OriGene, #SR308336, Rockville, MD, USA) using Lipofectamine RNAiMAX transfection reagent (Thermo Fisher Scientific, #13778030). Forty-eight hours later, cells were harvested using sample buffer containing 0.1 M Tris-HCl (pH 7.5), 4% SDS, 20% glycerol, 12% β-mercaptoethanol, and 1% bromophenol blue. For TCP1α knockdown, mouse primary cortical neurons were treated with TCP1α-siRNA (Sigma Aldrich, SASI_Mm01_00108559) or scrambled siRNA (OriGene, #SR30004). Forty-eight hours after transfection, cells were harvested and cytoplasmic/nuclear extracts were obtained (see “Subcellular fractionation”). For Erk1 and Erk2 knockdown, adult cortical neurons from 5xFAD mice at 1 month of age were transfected by Erk1-siRNA (Santa Cruz Biotechnology, sc-29308, Dallas, TX, USA), Erk2-siRNA (Santa Cruz Biotechnology, sc-35336, Dallas, TX, USA) or scrambled siRNA (OriGene, #SR30004). Forty-eight hours after transfection, cells were harvested using sample buffer containing 0.1 M Tris-HCl (pH 7.5), 4% SDS, 20% glycerol, 12% β-mercaptoethanol, and 1% bromophenol blue.

### Two-photon microscopy

This procedure has been described previously [25]. Adeno-associated virus 1 (AAV1)-EGFP with the synapsin I promoter (titer: 1 × 10^10^ vector genomes/mL, 1 µl) and AAV2-VAMP2-mCherry with the Cytomegalovirus (CMV) promoter were injected to two neighboring positions of retrosplenial cortex at −1.0 mm from bregma (mediolateral 0.5 mm; depth, 1 mm) and −3.0 mm from bregma (mediolateral 0.5 mm; depth, 1 mm), respectively, under anesthesia with 1% isoflurane. In the rescue experiment, AAV1-PQBP1 with the Cytomegalovirus (CMV) promoter (titer: 1 × 10^11^ vector genomes/mL, 1 µl) was injected together with AAV1-EGFP. After 3 weeks, the skull was thinned with a high-speed micro-drill in the mouse splenial cortex. Then, the head of each mouse was immobilized by attaching the head plate to a custom machine stage mounted on the microscope table. Two-photon imaging was performed using a laser-scanning microscope system FV1000MPE2 (Olympus, Tokyo, Japan) equipped with an upright microscope (BX61WI, Olympus, Japan), a water-immersion objective lens (XLPlanN25xW; numerical aperture, 1.05), and a pulsed laser (MaiTaiHP DeepSee, Spectra Physics, Santa Clara, CA, USA). EGFP and mCherry were excited at 920 nm and scanned at 495–540 nm and 575–630 nm, respectively. High-magnification imaging (101.28 μm × 101.28 μm; 1024 × 1024 pixels; 1 μm Z step) of the cortical layer I was performed with a 5 × digital zoom through the thinned-skull window in the retrosplenial cortex [25].

### Immunostaining and observation of transparent brains

PFA fixed cortical blocks of 5xFAD and control mouse brains were incubated in CUBIC 1 solution (Urea 25 wt%, Tetrakis ethylenediamine 25 wt%, Triton X 100 15 wt%) [26] for 48 h at 37 °C. Blocks were washed in PBS overnight at room temperature and then incubated with primary antibodies (pSer1068SRRM2 1:250; 6E10 1:400) in PBS with 0.2% Triton X 100, 1% BSA and 0.02% sodium azide for 48 h at 37 °C. Blocks were washed three times in PBS at room temperature for a total of 24 h and then incubated with secondary antibodies (goat anti mouse Alexa 488 and goat anti rabbit Alexa 647, 1:400) in PBS with 0.2% Triton X 100 and 0.02% sodium azide for 48 h at 37 °C. Blocks were washed three times in PBS at room temperature for 24 h and then incubated with SCALE S4 solution [27] at room temperature until imaging.

Images were acquired with a Carl Zeiss LSM 780 microscope equipped with a X40 c-Apochromat objective (numerical aperture, 1.2). Image processing was performed with Imaris Interactive Microscopy Image Analysis software (Bitplane, Zurich, Switzerland).

### Data analysis

Mass spectrum data of peptides were acquired and analyzed using Analyst TF (version 1.5, AB SCIEX). Based on the results, the corresponding proteins were retrieved from public databases of mouse and human protein sequences (UniProtKB/Swiss-Prot, downloaded from http://www.uniprot.org on June 22, 2010) using ProteinPilot (version 4, AB SCIEX, MA, USA), which uses the Paragon algorithm [28]. In ProteinPilot, tolerance of peptide search was set to 0.05 Da for MS and 0.10 Da for MS/MS analyses. “Phosphorylation emphasis” was set as sample description, and “biological modifications” was set at the processing specification step. The confidence score was used to evaluate the quality of the identified peptides, and the deduced proteins were grouped using the Pro Group algorithm (AB SCIEX, MA, USA) to eliminate redundancy. The threshold for protein detection in ProteinPilot was set at 95% confidence, and proteins with >95% confidence were accepted as identified proteins.

Quantification of peptides was performed by analysis of iTRAQ reporter groups in MS/MS spectra generated upon fragmentation in the mass spectrometer. For bias correction, signals of different iTRAQ reporters were normalized based on the assumption that the total signal of all iTRAQs should be equal. After bias correction, the ratio between reporter signals in 5xFAD and control mice (peptide ratio) was calculated.

The protein ratio was computed from the weighted average of peptide ratios corresponding to each protein; the peptide ratios were differentially weighted based on error factors after bias correction. The detailed formulas for calculation of these values are described in the AB SCIEX manual. In brief, after excluding peptides without an iTRAQ label, those sharing MS/MS data derived from other proteins, and those with low intensity, log values of iTRAQ ratios corresponding to a peptide were summed, and bias was divided by 10 and raised to the power of the sum. The result was treated as the quantity of the peptide.

For further data analyses, peptide and protein ratios were imported into Excel files from the ProteinPilot summaries. The quantity of a phosphopeptide fragment was calculated as the geometric mean of signal intensities of multiple MS/MS fragments including the phosphorylation site. Because the ratios of phosphoproteins and phosphopeptides obeyed a log-normal distribution, the ratios were transformed into logarithmic values to compare the 5xFAD and control groups. Because the data were postulated to be heteroscedastic, the difference between 5xFAD and controls was statistically evaluated by Welch’s test. P-values were adjusted using the Benjamini-Hochberg procedure.

### Analysis of Erk1/2 and Clk1 downstream pathways

The Erk1/2 downstream pathway was constructed using a PPI database compiled by the Genome Network project (http://genomenetwork.nig.ac.jp/index_e.html). Factors one or two degrees of separation from Erk1/2 (UniProt ID = Q63844 for Erk1 and P63085 for Erk2) in the database were selected as candidate nodes and edges in the Erk1/2 downstream pathway. Phosphorylation sites showing significant phosphorylation in the brains of 5xFAD mice were determined from phosphoproteome data by comparison of 5xFAD mice with B6/SJL mice. Whole cortex tissues from six 5xFAD mice and B6/SJL mice prepared at 1, 3, 6, and 12 months of age, were analyzed by mass spectrometry (Nano-LC to Triple TOF 5600 System, AB SCIEX); specimens from pooled B6 mice of the same age were used as controls. Phosphopeptides detected at >95% confidence were quantified relative to the control as described above, and values were integrated on specific phosphorylation sites. Proteins with detected phosphorylation site(s) were defined as detected phosphoproteins. The specificity of phosphorylation changes induced by Erk1/2 was tested by Fisher’s exact test of phosphorylation ratio in comparison with proteins that do not interact with Erk1/2.

### Analysis of exon skipping

For each gene, the expression values of exons were calculated by counting read numbers including the exon (described as ‘inclusion reads’). To quantify the degree of exon skipping, read numbers overlapping a splicing junction of two neighbors of each exon (described as ‘skipping reads’) were also counted. To detect exon splicing changes between untreated and PQBP1-cKO groups, skipping/inclusion ratios (the ratio of skipping and inclusion reads of an exon) of two groups were compared. Supplementary Tables 1, 3, and 4 show the Log2 values of the skipping/inclusion ratios of PQBP1-cKO or 5xFAD mice adjusted to the skipping/inclusion ratio of background mice. In all, a 2 × 2 contingency table was constructed using the numbers of skipping and inclusion reads, and Fisher’s exact test was used to determine statistical significance. P-values were adjusted using the Benjamini-Hochberg procedure. Adjusted p-values were denoted as q-values. Splicing changes were determined as significant at *q* < 0.05. For computing the exon skipping ratio correctly, each cell in the contingency table had to contain a positive integer.

### Gene ontology enrichment analysis

To create a list of genes for further functional analyses, changes in the levels of exon skipping were compared between PQBP1-cKO and control mice using Fisher’s exact test with the post-hoc Benjamini-Hochberg procedure. Significantly changed exons in terms of exon skipping were determined at *q* < 0.05 and used to select the corresponding genes. To specify the functions altered by the listed genes, Gene Ontology (GO) enrichment analysis was performed using the NIH Database for Annotation, Visualization, and Integrated Discovery (DAVID; https://david.ncifcrf.gov/). ‘Benjamini’ q-values calculated by DAVID were used for the threshold of enriched GO terms at *q* < 0.01. Representative functions of enriched GO terms were grouped by unsupervised hierarchical clustering in terms of commonly annotated genes. Distances between two groups were calculated using Ward’s method.

### Generation of a protein-protein interaction-based gene network

To generate a pathological gene network based on the altered gene functions in PQBP1-cKO, a list of genes annotated to significantly enriched GO term groups was created. UniProt IDs were added to the genes in the list. Genes whose UniProt IDs were not listed in the Human Genome Project (GNP) (http://genomenetwork.nig.ac.jp/index_e.html) database were removed from the list of genes. The selected genes were used for the generation of the pathological PPI network of the Synapsin-cKO mouse based on the integrated database of GNP including BIND (http://www.bind.ca/), BioGrid (http://www.thebiogrid.org/), HPRD (http://www.hprd.org/), IntAct (http://www.ebi.ac.uk/intact/site/index.jsf), and MINT (http://mint.bio.uniroma2.it/mint/Welcome.do). One additional edge and node were added to the selected nodes on the PPI database. A database of GNP-collected information was created on the Supercomputer System available at the Human Genome Center of the University of Tokyo.

### Analysis of gene network structure

The betweenness centrality score was used as the score of nodes in a network. A node with a high betweenness score can be interpreted as one that is connected with many nodes in terms of the information flow of paths. Therefore, it may have important roles in many biological pathways. The betweenness score was determined as ‘high’ at the top 25% of all genes.

### Statistics

A two-tailed t-test was used for comparisons of two groups. For comparison of multiple groups, Tukey’s HSD test was performed after one-way ANOVA. Statistical differences were determined significant at *p < 0.05 and **p < 0.01. Sample size was estimated based on preliminary experiments. Statistical analyses were performed with Microsoft Excel 2013 or R programming environment (version 3.4.0, R Foundation for Statistical Computing, Austria; available at https://www.R-project.org/).

### Behavioral test

Exploratory behavior was assessed using a Y-shape maze consisting of three identical arms with equal angles between each arm (O’HARA & Co., Ltd, Tokyo, Japan). Mice at the age of 6 months were placed at the end of one arm and allowed to move freely through the maze during an 8 min session. The percentage of spontaneous alterations (indicated as an alteration score) was calculated by dividing the number of entries into a new arm that was different from the previous one with the total number of transfers from an arm to another arm.

### Ethics

The experiments were approved by the Committees on Ethics, Gene Recombination Experiments, Human Ethics, and Animal Experiments of the Tokyo Medical and Dental University (O2014-005-03, 2010-215C3, 2011-22-3, and 0130225). The animal experiments were performed in strict accordance with the *Guidelines for Proper Conduct of Animal Experiments* by the Science Council of Japan.

## Results

### Phosphorylation of SRRM2 at Ser1068 precedes Aβ aggregate formation

**Fig. 1:**
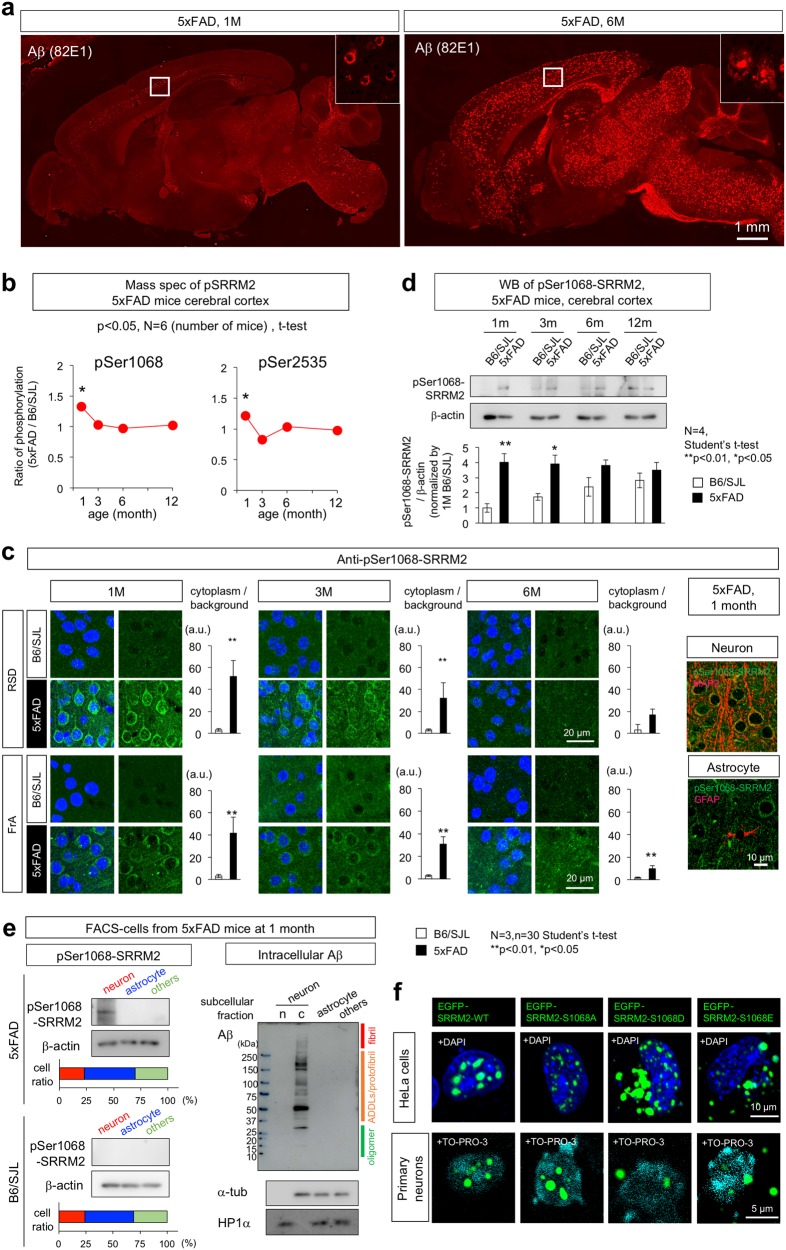
SRRM2 phosphorylation at pSer1068 was increased in 5xFAD mice. **a** Immunohistochemistry of 5xFAD mouse brains at 1 and 6 months of age with the most sensitive anti-Aβ antibody (82E1). No extracellular Aβ aggregates were detected, while intracellular Aβ accumulation was detected at 1 month of age. Extracellular Aβ aggregates were detected throughout the brain except cerebellum at 6 months of age. **b** Mass analyses were performed with whole cortex tissues from 5xFAD mice at 1, 3, 6, and 12 months of age. Phosphorylation was higher at two sites (Ser1068 and Ser2535) of SRRM2 at 1 month in 5xFAD mice than in the background B6/SJL mice (*N* = 3, *p < 0.05). **c** Immunohistochemistry of 5xFAD and B6/SJL mice at 1, 3, and 6 months of age revealed an increase of pSer1068-SRRM2 in neurons. Cytoplasmic staining of neurons was ubiquitously detected in the retrosplenial dysgranular cortex (RSD) and frontal association cortex (FrA) in 5xFAD mice at 1 month, whereas the signals were lower at later ages. The other brain regions are shown in Supplementary Fig. 2. Quantitative analyses of intensities are shown in graphs at each time point and in each area. Signal intensities were determined in six cytoplasmic areas of a single cell, and the mean was adjusted to the background intensity. The corrected mean values from 30 cells in each brain area were used to calculate the representative value of a mouse. Statistical analysis was performed with the values of three mice in each area at each time point by Student’s *t*-test. Right panels show co-staining of pSer1068-SRRM2 with MAP2 or GFAP. **d** Western blot analyses of mouse cortex tissues at 1, 3, 6, and 12 months confirmed the increase at 1 month and subsequent decline of pSer1068-SRRM2 in 5xFAD mice. **e** FACS-sorted cells from cerebral cortex of 5xFAD mice at 1 month were blotted with anti-pSer1068-SRRM2 antibody (left panels) or anti-Aβ antibody (82E1) (right panels). The ratios of neurons (MAP2-positive), astrocytes (GFAP-positive) and other cells (MAP2-negative and GFAP-negative) are shown in lower graph. Western blot with anti-Aβ antibody revealed that intracellular Aβ already formed ADDLs or protofibrils and a small part of reached to the fibril state. **f** Intracellular localization of EGFP-tagged phosphorylation mimicry mutants (S1068A, S1068D, and S1068E) of SRRM2 in Hela cells and primary cortical neurons prepared from E15 mouse embryos 48 h after transient transfection

Changes of SRRM2 phosphorylation at multiple sites were examined during aging in 5xFAD mice carrying APP transgenes containing the Swedish (K670N, M671L), Florida (I716V), and London (V717I) mutations and PS1 with two FAD mutations, M146L and L286V [20]. This mouse model was the same as that used in our previous comprehensive phosphoproteome analyses [1]. Phosphorylation changes were detected at 10 sites by mass spectrometry analyses of whole cortex tissues prepared from 5xFAD mice at 1, 3, 6, and 12 months of age. Consistently with our previous results with the 82E1 and 6E10 anti-Aβ antibodies [1], we reconfirmed that extracellular Aβ aggregate was not detected even by the most sensitive 82E1 anti-Aβ antibody at the earliest time point at 1 month of age (Fig. 1a). At this age, phosphorylation was increased at two sites (Ser1068 and Ser2535) in SRRM2 (Fig. 1b).

We next investigated whether neurons were responsible for the phosphorylation of SRRM2 by generating antibodies against phosphopeptides corresponding to two phosphorylation sites, and performed immunohistochemistry of 5xFAD mice. At 1 month of age, anti-pSer1068-SRRM2 polyclonal antibody detected cytoplasmic staining of cortical neurons (Fig. 1c), whereas anti-pSer2535 antibody did not reveal any remarkable differences (data not shown). We confirmed identities of stained cells by neuronal and glial markers (Fig. 1c, right panels). Reactivity to the anti-pSer1068-SRRM2 polyclonal antibody was confirmed (Supplementary Fig. 1). pSer1068 positive neurons were broadly distributed in the frontal cortex (FrA), motor cortex (M2), occipital cortex (V2) and parietal cortex (RSD), but not in hippocampus (CA1), cerebellum or brain stem (Fig. 1c, Supplementary Fig. 2). Phosphorylation at Ser1068 decreased at later stages (3 and 6 months of age) (Fig. 1c). Western blot analyses confirmed the increase of pSer1068-SRRM2 at 1 month of age and the subsequent decline (Fig. 1d).

The signal intensity per neuron determined by immunohistochemistry (Fig. 1c) showed a greater increase than that determined by mass spectrometry analysis (Fig. 1b) or by western blot that also exaggerated the increase of pSer1068-SRRM2 (Fig. 1d); this might be attributed to the exclusion of the dilution of the increased signal of neurons by non-neuronal cells. Therefore, we separated cell types by fluorescence activated cell sorting (FACS) from cerebral cortex of 5xFAD and B6/SJL mice at 1 month of age, and performed western blot (Fig. 1e). The percentage of neuron; glia; others were grossly 25; 45; 30%. The band signal of pSer1068-SRRM2 corrected by β-actin in neuron, glia and others fractions were 0.26; 0.027; 0.018 (Fig. 1e, left panels). Calculation with these data supported 4 to 5 folds increase of pSer1068-SRRM2 in cortex tissues. The discrepancy between western blot and mass analysis is unknown while the issue has been already generally recognized (http://www.asbmb.org/asbmbtoday/asbmbtoday_article.aspx?id=48201). In our case, reliability for peptide detection in mass analysis was more than 95%, which is far higher than most proteome reports, might have set the threshold higher and decreased the sensitivity. Surprisingly, western blot with anti-Aβ antibody using the same FACS-sorted cells revealed that most of intracellular Aβ already formed ADDLs or protofibrils and a small part of reached to the fibril state at 1 month of age (Fig. 1e, right panels).

These results consistently suggested that further investigation of SRRM2 phosphorylation at Ser1068 might shed light on the pre-clinical and pre-aggregation stages of AD pathology.

### Phosphorylation at Ser1068 changes the subcellular localization of SRRM2

Next, we examined whether Ser1068 phosphorylation affects the subnuclear or subcellular localization of SRRM2. First, EGFP-tagged phosphorylation site mutants mimicking phosphorylated (S1068D and S1068E) and non-phosphorylated SRRM2 (S1068A) were generated. Phosphorylation-mimetic EGFP-SRRM2 mutants were distributed predominantly in the cytoplasm of HeLa cells, whereas the non-phosphorylated SRRM2-mimetic mutant maintained a predominantly nuclear distribution (Fig. 1f). The cytoplasmic shift of phosphorylation-mimetic EGFP-SRRM2 mutants (S1068D and S1068E) was also observed in primary cortical neurons prepared from E15 mouse embryos (Fig. 1f).

To distinguish endogenous localization of SRRM2 phosphorylated and non-phosphorylated at Ser1068, we employed another antibody (ab122719) whose antigen region overlaps with that of anti-pSer1068-SRRM2 antibody (Supplementary Fig. 3a). The ab122719 antibody detected nuclear SRRM2 corresponding to non-phosphorylated EGFP-SRRM2-WT while anti-pSer1068-SRRM2 antibody detected cytoplasmic SRRM2 corresponding to mutants mimicking phosphorylated SRRM2 (Supplementary Fig. 3b).

By using the two antibodies, we found that SRRM2 is phosphorylated at Ser1068 under stress such as high osmolarity and localized to the cytoplasm (Supplementary Fig. 3c). When 0.4 M sorbitol was applied to culture medium of HeLa cells (750 mOsm/L) for 60 min, nuclear non-phosphorylated SRRM2 was decreased and cytoplasmic pSer1068-SRRM2 was increased (Supplementary Fig. 3c). We also suspected that pSer1068-SRRM2 might be localized to P-bodies or other stress granules. However, stress granule stained by TIA [29], P-body stained by GW182 [30] and nuclear stress granule stained by HSF1 [31] did not include pSer1068-SRRM2 in co-staining (Supplementary Fig. 3c).

### Phosphorylation at Ser1068 prevents SRRM2 from interacting with TCP1

**Fig. 2:**
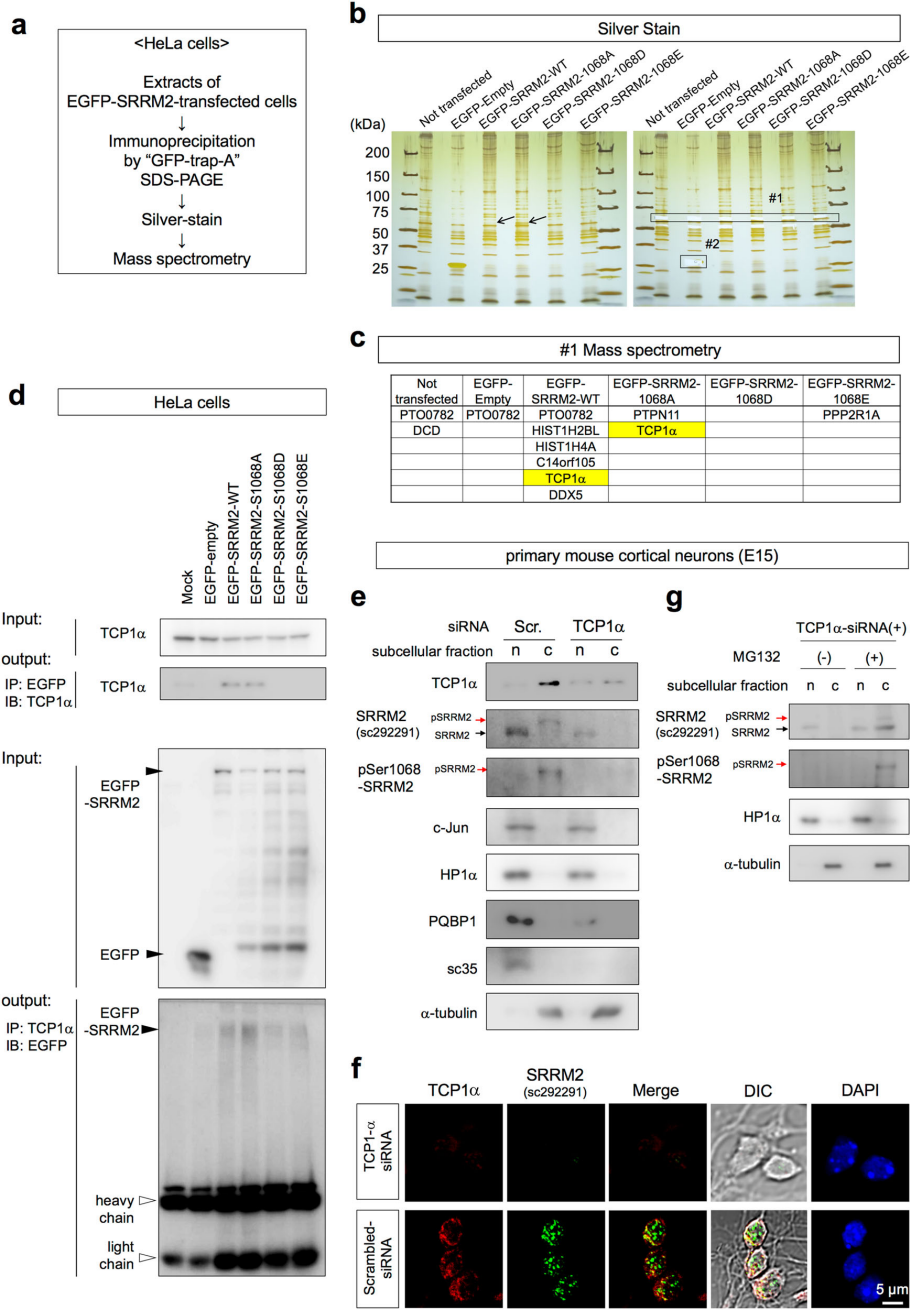
pSer1068 prevents interaction with TCP1α and decreases SRRM2. **a** Experimental procedure to detect binding proteins specific to phosphorylated and/or non-phosphorylated SRRM2. **b** A specific band was detected in the precipitate of the non-phosphorylated SRRM2 mimicry mutant (1068 A) and more faintly in the precipitate of the wild-type SRRM2. #1 and #2 were cut out from the gel and subjected to mass spectrometry. **c** Results of mass spectrometry of the bands cut out from the silver stained gel. **d** Phosphorylated/non-phosphorylated SRRM2 mimicry mutants were transiently expressed in HeLa cells, and interaction with endogenous TCP1α was tested by immunoprecipitation. **e** Primary cortical neurons prepared from mouse E15 embryos were transiently transfected with TCP1α or scrambled siRNA, and multiple protein levels in the nucleus and cytoplasm fractions were evaluated by western blot analysis. **f** Immunocytochemistry of primary neurons confirmed that TCP1α-KD decreased SRRM2. **g** The TCP1α-KD-induced reduction of the SRRM2 protein was inhibited by addition of MG132 to the culture medium

To understand the molecular mechanism underlying the change of subcellular localization of SRRM2, proteins binding to wild-type and phosphorylation site mutants of SRRM2 were screened in HeLa cells (Fig. 2a). Pull-down purification of EGFP-tagged wild-type and phosphorylation site mutants of SRRM2 was followed by analysis of co-precipitated proteins by mass spectrometry (Fig. 2a). Silver stains of PAGE-separated co-precipitated proteins showed a 55 kD band that appeared only in wild-type and the non-phosphorylated SRRM2 mimicry mutant (S1068A) (Fig. 2b, #1). A band at the size of the EGFP protein (Fig. 2b, #2) was analyzed by mass spectrometry to confirm the soundness of the method.

The mass analysis showed that T-complex protein 1 subunit alpha (TCP1α) co-precipitated with wild-type SRRM2 and the non-phosphorylated SRRM2 mimicry mutant (S1068A) (Fig. 2c). Immunoprecipitation further confirmed the interaction between TCP1α and the wild type or the S1068A mutant, although the affinities of the phosphorylation-mimetic SRRM2 mutants (S1068D, S1068E) for TCP1 were diminished (Fig. 2d).

Considering the well-known function of the TRiC complex containing TCP1α as a cytoplasmic Group II chaperonin for folding newly synthesized proteins from the endoplasmic reticulum (ER) and sorting them to appropriate cellular domains, these results indicated that the deficient interaction between pSer1068-SRRM2 and TCP1α impairs nuclear transport.

### Loss of interaction with TCP1α disables nuclear transport of SRRM2

To test whether interaction with TCP1α is essential for the nuclear transport of SRRM2, we examined the effect of siRNA-mediated TCP1α knockdown on endogenous SRRM2 protein levels in the nuclear and cytoplasmic fractions of primary neurons (Fig. 2e). TCP1α knockdown markedly downregulated nuclear SRRM2, whereas it had no effect on the levels of nuclear c-Jun or HP1α protein or cytoplasmic α-tubulin (Fig. 2e). In mouse cortical neurons, pSer1068-SRRM2 was detected in the cytoplasmic fraction, which could be attributed to the stressful conditions of primary culture. Cytoplasmic pSer1068-SRRM2 was also decreased but not completely eliminated by TCP1α knockdown (Fig. 2e). Several proteins such as PQBP1 and SC35 were downregulated (possible reasons will be discussed later), whereas the nuclear or cytoplasmic levels of other proteins such as c-Jun or HP1α did not change (Fig. 2e). The results of immunocytochemistry of mouse primary cortical neurons supported the TCP1α knockdown-induced downregulation of SRRM2 (Fig. 2f).

Addition of the proteasome inhibitor MG132 prevented the decrease of cytoplasmic pSer1068-SRRM2 in response to TCP1α deficiency (Fig. 2g), suggesting that loss of interaction with TCP1α resulted in unfolding of SRRM2 protein in the cytoplasm, its degradation by the proteasome system, and loss of its transfer to the nucleus. Thus, increased phosphorylation at Ser1068 would be expected to downregulate both nuclear and cytoplasmic SRRM2. MG132 increased non-phosphorylated SRRM2 in the cytoplasm (Fig. 2g), suggesting that, in the absence of TCP1α, SRRM2 cannot be folded and transferred to the nucleus and that proteasome system also degrades such unfolded non-phosphorylated-SRRM2 in the cytoplasm.

### Deficiency of nuclear SRRM2 destabilizes PQBP1

**Fig. 3:**
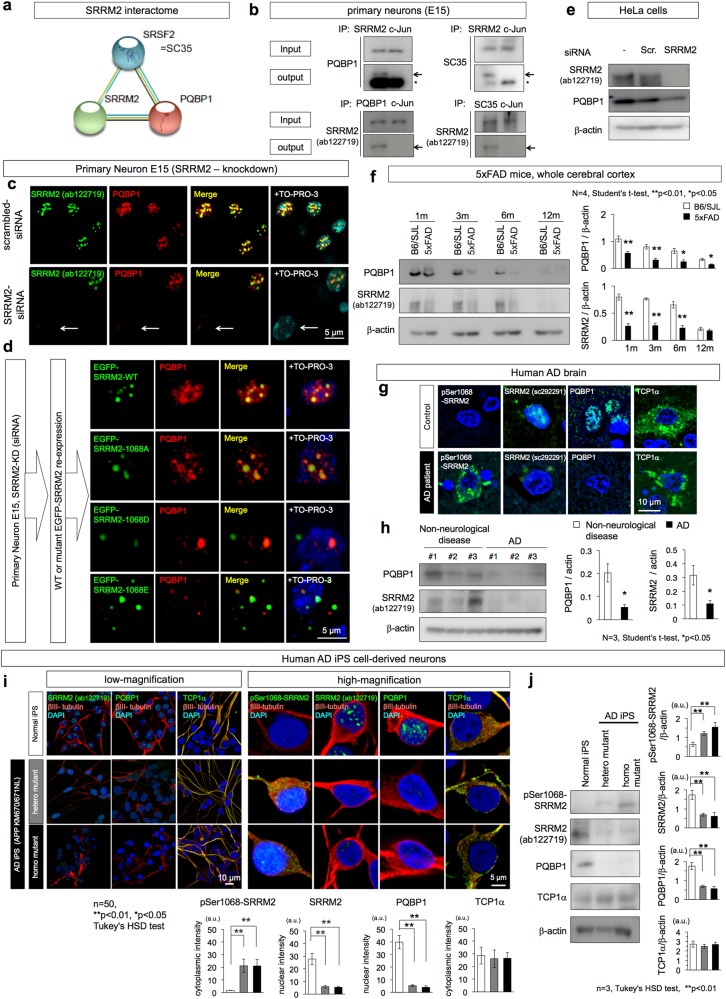
SRRM2 deficiency destabilizes PQBP1. **a** Interactome database (String ver.10.5) predicted the interaction of PQBP1 and SC35 with SRRM2. **b** Immunoprecipitation revealed the interaction between endogenous SRRM2 and PQBP1 or between SRRM2 and SC35. **c** SiRNA-mediated SRRM2 knockdown reduced PQBP1 in primary cortical neurons prepared from E15 mouse embryos (white arrow). **d** Expression of EGFP-wild-type or 1068A-mutant SRRM2 recovered PQBP1, whereas EGFP-1068D-mutant or 1068E-mutant SRRM2 did not recover PQBP1 sufficiently. **e** Western blot analysis confirmed SRRM2 knockdown by siRNA and the accompanying decrease of PQBP1 in HeLa cells. **f** Western blot analysis of whole cerebral cortex lysates from 5xFAD mice and sibling mice (B6/SJL) at each age revealed that PQBP1 decreased during aging and the decrease occurred more rapidly in 5xFAD mice. **g** Immunohistochemistry was performed with parietal lobe tissues of human AD patients with PS1 Met146Leu mutation (*N* = 3) and non-neurological disease controls (*N* = 3). Reduction of SRRM2 and PQBP1 was obvious in AD patients, and representative images of cortical neurons are shown. **h** Western blot analysis was performed with the same AD and non-neurological disease patients. The right graphs show the quantitative analysis of β-actin-corrected PQBP1 and SRRM2 signals (*N* = 3, *p < 0.05, Student’s *t*-test). **i** Human iPS cells carrying the APP KM670/671NL heterozygous or homozygous mutation generated by Crispr-cas9 based genome editing were used to show that SRRM2 and PQBP1 are lower in the nuclei and that pSer1068-SRRM2 is increased in the cytoplasm of differentiated neurons. Cytoplasmic levels of TCP1α in human iPS cells carrying the APP KM670/671NL mutation and in human normal iPS cell-derived neurons were similar. **j** Western blot analysis of neurons differentiated from human iPS cells carrying the APP KM670/671NL heterozygous or homozygous mutation. Right panels show quantification of the band intensities

Interacting partners of SRRM2 that may affect neuronal functions were searched in the protein-protein interaction (PPI) database with String ver. 10.5 (URL: https://string-db.org/cgi/network.pl?taskId=Xp6fbEO1skQv). The PPI database identified an interaction between SRRM2 and PQBP1 (Fig. 3a), a causative gene for ID [7], and the interaction was confirmed by immunoprecipitation (Fig. 3b).

Next, we examined the localization of SRRM2 and PQBP1 in the nucleus (Supplementary Fig. 4). In HeLa cells, SRRM2 and PQBP1 co-localized at small speckles in the nucleus under physiological conditions (Supplementary Fig. 5a, upper panels). The speckles included SC35 (Supplementary Fig. 5b, lower panels), which was predicted to interact with PQBP1 by String ver. 10.5 (https://string-db.org/cgi/network.pl?taskId=ovqHiNBbIVhv) (Fig. 3a). The interaction between SRRM2 and SC35 was also confirmed by immunoprecipitation (Fig. 3b).

siRNA-mediated knockdown of SRRM2 substantially downregulated PQBP1 both in HeLa cells and mouse cortical primary neurons (Fig. 3c, Supplementary Fig. 5a). PQBP1 was hardly detected in siRNA-transfected neurons (white arrow in Fig. 3c) while non-transfected neurons in the same image did not show the decrease of PQBP1 protein (Fig. 3c). Re-expression of wild-type and 1068 A mutant SRRM2 recovered PQBP1 speckles in the nucleus of primary neurons after siRNA-meditated SRRM2 knockdown (Fig. 3d), whereas phosphorylation-mimetic SRRM2 mutants (S1068D, S1068E) did not recover PQBP1 appreciably (Fig. 3d). Western blot analysis confirmed the SRRM2-KD-related downregulation of PQBP1 (Fig. 3e).

SRRM2-KD induced decrease of PQBP1 also in HeLa cells (white arrow in middle panels of Supplementary Fig. 5a). Re-expression of EGFP-SRRM2 recovered PQBP1 in SRRM2-siRNA-transfected cells (green arrow in Supplementary Fig. 5a) whereas the recovery was not found in cells without EGFP-SRRM2 (white arrow in Supplementary Fig. 5a). The similar decrease of SC35 associated with SRRM2-KD was confirmed in HeLa cells (Supplementary Fig. 5b).

To determine whether the changes of SRRM2 and PQBP1 take place in AD pathology, cerebal cortex samples from 5xFAD mice and human AD patients were analyzed by western blot and immunohistochemistry (Fig. 3f–h). In mouse samples, PQBP1 and SRRM2 were decreased in cerebral cortex of 5xFAD mice (Fig. 3f). Consistently, immunohistochemistry of human postmortem AD brains revealed reduction of SRRM2 and PQBP1 in the nucleus in addition to increase of pSer1068-SRRM2 in the cytoplasm (Fig. 3g). Western blot analyses of temporal lobe samples from human AD patients supported the decrease of SRRM2 and PQBP1 (Fig. 3h).

Moreover, immunohistochemistry and western blot analysis of pan-neurons differentiated from human iPS cells in which the APP gene was mutated (*APP KM670/671NL)* by genome editing also supported the reduction of SRRM2 and PQBP1 together with the increase of pSer1068-SRRM2 in human AD neurons (Fig. 3i, j). Meanwhile. TCP1α was not changed in iPS-derived AD neurons (Fig. 3i, j).

### Loss of PQBP1 impairs synapses via RNA splicing of synapse genes

To investigate the effect of PQBP1 deficiency in vivo, we generated a mature neuron-specific *Pqbp1* conditional knockout (cKO) mouse by crossing previously reported *Pqbp1*-loxP mice [16] with *Synapsin*-Cre mice. The resultant *Pqbp1*-cKO mice were subjected to synapse morphology analysis by two-photon microscopy and expression profiling of synapse-related genes by RNA-sequencing analysis using a next generation sequencer.

**Fig. 4:**
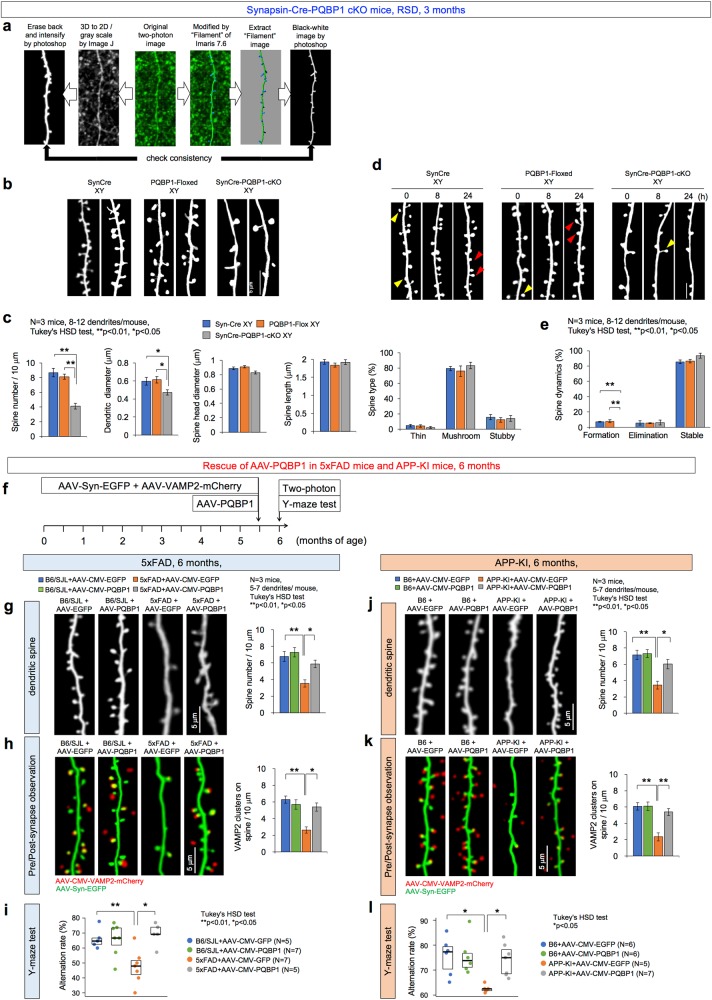
PQBP1 rescues synapse and cognitive function in AD mouse models. **a** Image processing used for quantification of spine numbers and figure presentation. Leftward process is generally used for generating spine images, while rightward process was employed in this study to quantify spine numbers and other parameters. Both of the final images were checked in regard of the spine identity. **b** Two-photon microscopic observation of dendritic spines in Synapsin-Cre male mice (SynCre XY), PQBP1-Floxed male mice (PQBP1-Floxed XY), and Synapsin-Cre PQBP1-cKO male mice (Syn-Cre PQBP1-cKO XY) are shown. **c** Quantitative analysis (3 mice in each group, 8–12 dendrites/mouse, Tukey’s HSD test) revealed that dendritic spines (protrusions) were lower in PQBP1-deficient mature neurons. **d** Live-imaging of dendritic spines with two-photon microscopy was performed for 24 h in three genotypes of mice. Newly formed spines are indicated with red arrowheads, eliminated spines are indicated with yellow arrowheads. **e** Quantitative analyses of dendritic protrusions revealed that formation of spines was lower in Synapsin-Cre PQBP1-cKO mice (3 mice in each group, 8–12 dendrites/mouse, Tukey’s HSD test). **f** AAV-PQBP1 mediated rescue of synapse pathology in 5xFAD male and APP-KI male mice. The mice received a single injection of AAV-PQBP1 into RSD at 5.5 months and were evaluated 2 weeks later at 6 months of age. **g** Two-photon microscopic images of dendritic spines in the first layer of RSD in 5xFAD or B6/SJL male mice after injection of AAV-EGFP or AAV-PQBP1. The right graph shows the quantitative analysis of spine number. **h** Two-photon microscopic images of contact of axon terminals and dendritic spines in the first layer of RSD in 5xFAD male mice after injection of AAV-Vamp-Cherry with AAV-EGFP or AAV-PQBP1. The right graph shows the quantitative analysis of the axon terminals merged on the spine. **i** Alteration ratios in the Y-maze test of 5xFAD male mice after injection of AAV-EGFP or AAV-PQBP1 are shown. **j**. Two-photon microscopic images of dendritic spines in the first layer of RSD in APP-KI or B6 male mice after injection of AAV-EGFP or AAV-PQBP1. The right graph shows the quantitative analysis of spine number. **k** Two-photon microscopic images of contact of axon terminals and dendritic spines in the first layer of RSD in APP-KI or B6 male mice after injection of AAV-Vamp-Cherry with AAV-EGFP or AAV-PQBP1. The right graph shows the quantitative analysis of the axon terminals merged on the spine. **l** Alteration ratios in the Y-maze test of APP-KI male mice after injection of AAV-EGFP or AAV-PQBP1 are shown

First, dendritic spine morphologies in vivo were obtained from two-photon microscopic observation of layer 1 of the retrosplenial dysgranular cortex (RSD) in *Pqbp1*-cKO mice at 3 months of age by using the image analysis methods (Fig. 4a). Deficiency of Pqbp1 in neurons reduced the number of dendritic spines, whereas it did not affect the diameter or length of spines (Fig. 4b, c). Consistently, the morphology of dendritic spines, which is characterized as thin, mushroom, or stubby, was unaltered in *Pqbp1*-cKO mice (Fig. 4c). Position-fixed chronological observation at multiple time points revealed that dendritic spine synthesis (formation) was decreased (Fig. 4d, e), explaining the reduction of dendritic spines.

Cerebral cortex tissues of *Pqbp1*-cKO mice at 3 months of age were subjected to RNA sequencing to investigate the effect of Pqbp1 deficiency on gene expression and RNA splicing profiles. Changes in gene expression and splicing pattern were analyzed using RPKMs and exon skipping ratios (Supplementary Fig. 6a) as described previously [16, 32]. Genes containing an exon in which the skipping ratio differed significantly between Wt and *Pqbp1*-cKO mice (*q* < 0.05 in Fisher’s exact test with post-hoc BH procedure) were selected (Supplementary Fig. 6a) and used for further bioinformatics analyses.

Gene ontology (GO) enrichment analysis of the selected genes was performed (Supplementary Fig. 6b), and the enriched GO term groups were connected if two groups shared common genes (Supplementary Fig. 6c). The data strongly suggested that PQBP1 deficiency in mature neurons has a marked effect on synapse-related functions. Next, a pathological gene network was generated based on the integrated protein-protein interaction database (http://genomenetwork.nig.ac.jp/index_e.html) with the selected genes and the one-step edges (Supplementary Fig. 6d). We further searched for core genes in the pathological network based on the betweenness score (Supplementary Fig. 6e). The top three core genes with the highest betweenness scores were GAPDH, Ywhae (14-3-3 ε), and hnRNPK (Supplementary Fig. 6e). This result was consistent with the comprehensive phosphoproteome analysis suggesting that GAPDH is a core protein in 5xFAD mice [1] and with the role of 14-3-3 ε in critical synaptic functions [33, 34]. Mutations of hnRNPK were associated with AU-Kline syndrome associated with ID [35, 36]. Interestingly, APP was ranked at the 5th position, suggesting its importance in the PQBP1-regulated network. Taken together, these data indicated that PQBP1 is a key regulator of synapse function via its ability to modulate RNA splicing.

### AAV-PQBP1 rescues synapses and the phenotype of AD model mice

Based on the hypothesis that PQBP1 downregulation in neurons caused synapse dysfunction in the AD models, we tested whether PQBP1 supplementation could recover the decrease of spine density by increasing the expression of synapse related genes. In parallel, we tested how PQBP1 supplementation could rebuild contact of pre- and post-synaptic components by developing a new method (Fig. 4f). The number of dendritic spines at 6 months of age recovered when AAV-PQBP1 was injected into the RSD of 5xFAD mice at 2 weeks before two-photon microscopic observation (Fig. 4f, g). In addition, AAV-PQBP1 recovered the number of axon terminals in contact with a spine, as visualized by VAMP2-mCherry (Fig. 4h). Mature synapses, in which the axon terminal and spine were merged, were decreased in 5xFAD mice and recovered by AAV-PQBP1 injection (Fig. 4h). Consistent with the recovery of synapse morphology, the score in the Y-maze test improved in response to AAV-PQBP1 supplementation (Fig. 4i).

The same set of experiments was performed with “female” 5xFAD mice at the same age (Supplementary Fig. 7a). The number of dendritic spines was decreased in female 5xFAD mice to a lesser extent, but similarly recovered by AAV-PQBP1 (Supplementary Fig. 7b). Mature synapses and cognitive function in Y-maze test were also mildly decreased in female 5xFAD mice and recovered by AAV-PQBP1 (Supplementary Fig. 7c, d).

Moreover, similar experiments were performed with APP-KI mice (*App*^*NL-G-F/NL-G-F*^), which confirmed the rescue effect of AAV-PQBP1 on dendritic spine density (Fig. 4j) and mature synapse (Fig. 4k) in APP-KI mice. Consistently, the abnormal decline of cognition observed in APP-KI mice in the Y-maze test recovered to normal levels in the presence of AAV-PQBP1 at 24 weeks of age (Fig. 4l).

RNA sequencing analysis of PQBP1-cKO mice and 5xFAD mice revealed that 103 exons in 47 genes showing changes of RNA splicing were shared (Supplementary Fig. 8, Supplementary Table 1). Among them, 41 exons (40%) were recovered by AAV-PQBP1 in 5xFAD mice, which included exons of synapse-related genes such as G protein-coupled receptor associated sorting protein 1 (Gprasp1), Neurogranin (Nrgn), and Synaptosome associated protein 25 (Snap25) (Supplementary Table 2). Interestingly, skipping of exon 8 in MEK1 gene was increased (Supplementary Table 2). It is of note that cancer mutations of Erk1 gene that generally increase Erk1 activity involve G301X mutation in exon 8 associated with lung adenocarcinoma [37].

### Erk1/2 phosphorylate SRRM2 at Ser1068

**Fig. 5:**
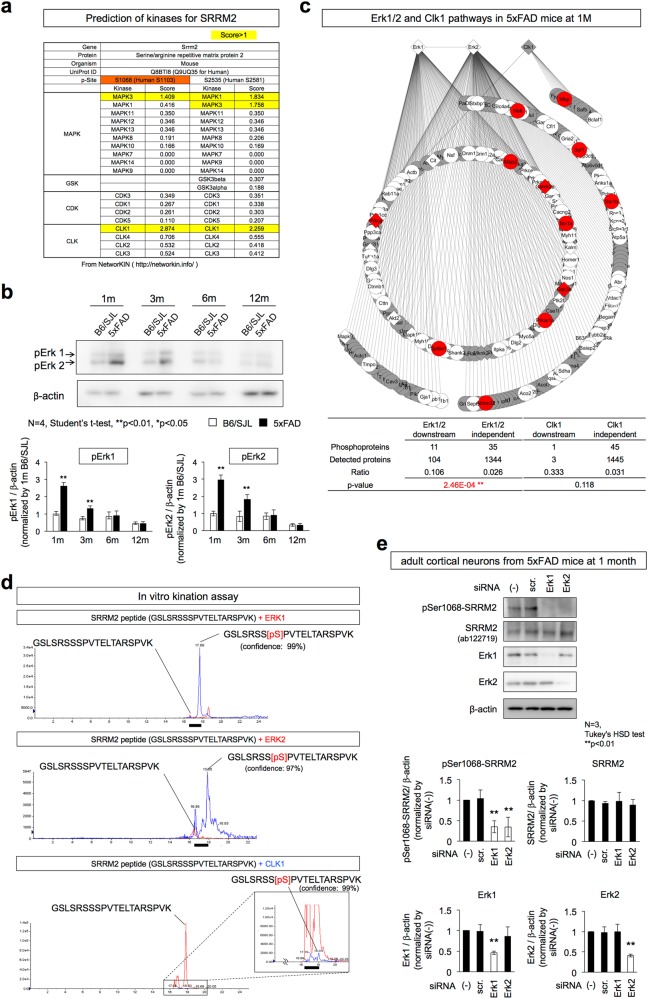
Erk1/2 phosphorylates SRRM2 at Ser1068. **a** Kinase prediction by NetworKIN (http://networkin.info/). Erk1/MAPK3 and Clk1 showed scores of >1.0 for Ser1068 of mouse SRRM2 and the counterpart Ser1103 of human SRRM2. **b** Western blot detection of Erk1/2 (MAPK3/1) in the cerebral cortex of 5xFAD mice from 1 to 12 months of age. The lower graph shows the results of quantitative analyses. **c** Downstream target proteins of Erk1/2 but not of Clk1 are activated in 5xFAD mice. Proteins shown in red indicate increased phosphorylation at 1 month of age in whole cerebral cortex tissues of 5xFAD mice. White: detected by mass analysis but unchanged, red: detected and increased significantly in Welch’s test with post-hoc BH procedure (*p* < 0.05), gray: undetected by mass analysis. Diamond: kinase protein, circle: non-kinase protein. In lower panel, the ratio of phosphorylated proteins / total proteins was compared between inside and outside of kinase downstream by Fisher’s exact test (*p* < 0.05). The high quality figure is posted at http://suppl.atgc.info/021/. **d** To confirm that these activated kinases are responsible for SRRM2 phosphorylation at Ser1068, in vitro phosphorylation was performed with SRRM2 peptide substrate (GSLSRSSSPVTELTARSPVK) and Erk1/MAPK3, Erk2/MAPK1, or Clk1 was purified followed by detection of phosphorylated substrates by mass spectrometry. The peaks in LC that were identified by MS/MS as phosphorylated or non-phosphorylated SRRM2 peptides are indicated. **e** SiRNA-mediated knockdown of Erk1 or Erk2 suppresses SRRM2 phosphorylation at Ser1068. Lower graphs show quantitative analyses of band signal intensities from three mice (*N* = 3). Double asterisks indicate *p* < 0.01 in Tukey’s HSD test

Candidate kinases that could phosphorylate SRRM2 at Ser1068 were searched using NetworKIN (http://networkin.info/). The scores of Erk1/MAPK3 and Clk1 were selectively higher, reaching functional levels in confirmed cases (Fig. 5a). Western blot analysis showed that the active forms of MAPK1/3 (Erk2/1) were increased at 1 and 3 months of age in 5xFAD mice (Fig. 5b). Pathway analysis of phosphorylated proteins in whole cerebral cortex tissues of 5xFAD mice supported the increased phosphorylation of downstream target proteins of Erk1/2 but not those of Clk1 at 1 month of age (Fig. 5c). The number of Clk1 downstream proteins is small, and *p*-value was not sufficient to support the significance (Fig. 5c). Activation of Erk1/2 downstream pathways was attenuated after 3 months of age (Supplementary Fig. 9). To confirm that these activated kinases were responsible for SRRM2 phosphorylation at Ser1068, we performed in vitro phosphorylation assays using a SRRM2 polypeptide substrate (GSLSRSS**S**PVTELTARSPVK) that included Ser1068, and purified Erk1/MAPK3, Erk2/MAPK1, or Clk1 followed by detection of phosphorylated substrates by mass spectrometry (Fig. 5d). The results showed that Erk1/2 but not Clk1 efficiently phosphorylated the SRRM2 polypeptide substrates including those with Ser1068 (Fig. 5d). We also performed Erk1/2-KD in adult cortical neurons at 1 months of age, and confirmed that Erk1/2-KD prevented phosphorylation of SRRM2 (Fig. 5e). These results collectively indicated that Erk1/2 phosphorylates SRRM2 at Ser1068.

### Intracellular Aβ is associated with SRRM2 phosphorylation at Ser1068

Finally we investigated the relationship between Aβ and SRRM2 phosphorylation at Ser1068. Confocal microscopic observation of transparent brains of 5xFAD mice and the corresponding 3D images revealed no definite relationship between extracellular Aβ and pSer1068-SRRM2 stained cells (Supplementary Video). Therefore, to investigate the relationship between intracellular Aβ and SRRM2 phosphorylation at Ser1068, we next performed double staining of brains from 5xFAD mice, APP-KI mice (*App*^*NL-G-F/NL-G-F*^) and human AD patients carrying a PS1 mutation (Met146Leu). Cortical neurons accumulating intracellular Aβ were stained with pSer1068-SRRM2 in all cases (Supplementary Fig. 10a-c). We confirmed that these intracellular Aβ-positive cells were neurons but not microglia or astrocytes (Supplementary Fig. 10d). In neurons accumulating intracellular Aβ, pSer1068-SRRM2 was increased, SRRM2 and PQBP1 were decreased (Supplementary Fig. 11, 12), and Erk1/2 were activated (Supplementary Fig. 13) consistently, suggesting that intracellular accumulation of Aβ activates Erk1/2 to induce SRRM2 phosphorylation at Ser1068 and the subsequent decrease of SRRM2 and PQBP1.

The increase of pSer1068-SRRM2 in cortical neurons of 5xFAD mice, APP-KI mice and human PS1-linked AD patients was not directly related to extracellular Aβ aggregates (i.e., the distance from extracellular Aβ aggregates did not determine the level of pSer1068-SRRM2 in neurons) (Supplementary Fig. 14). Similarly, the increase of pErk1/2 in cortical neurons of 5xFAD mice, APP-KI mice and human PS1-linked AD patients was not directly related to the distance from extracellular Aβ aggregates (Supplementary Fig. 15).

It is of note that we frequently detected cytoplasmic aggregate-like dots of pSer1068-SRRM2 in cortical neurons of human PS1-linked AD patients, in which and Aβ were co-localized (Supplementary Fig. 10). Interestingly, SRRM2 is a typical intrinsically disordered/denatured protein (http://iupred.enzim.hu), The data might suggest a close relationship between SRRM2 and intracellular Aβ.

## Discussion

### PQBP1 mediates the pre-aggregation pathology of Alzheimer’s disease

In this work, we showed that phosphorylation of SRRM2 at Ser1068 before histological Aβ aggregation in the AD model mice changed the subcellular localization of SRRM2 from the nucleus to the cytoplasm. We also showed that this shift in SRRM2 localization downregulated the splicing-regulatory protein PQBP1, which in turn widely affected pre- and post-synapse proteins, leading to morphological changes of synapses in the cerebral cortex. PQBP1 supplementation recovered not only synapse morphology but also cognitive impairment in the AD model mice. Our results indicated that SRRM2 and PQBP1 levels were slightly lower in the cerebral cortex of postmortem human AD patients, and Erk1/2 were responsible for the phosphorylation of SRRM2 at Ser1068. These results collectively suggest a new concept that splicing-related proteins play critical roles in the pathology of AD at the early stage.

General impairment of RNA splicing and/or gene expression profiles has been implicated in frontotemporal lobal degeneration (FTLD), another group of neurodegenerative dementias, since aggregated proteins in FTLD such as TDP43 and FUS are involved in RNA splicing. However in AD, although the specific impairment of tau or ApoE receptor-2 is known [38], general impairment of RNA splicing has not been established as a pathological domain. A pioneering work suggesting the involvement of RNA splicing in AD was reported by Bai et al. [39]. These authors analyzed the human brain-insoluble proteome in AD by mass spectrometry and identified multiple U1 snRNP subunits in cytoplasmic aggregates, concluding that the effect on RNA splicing of tau is a major cause of neurodegeneration. Although different models and proteins were used, we found that, in contrast to their findings showing that RNA splicing of tau occurs during or after the formation of protein aggregates, phosphorylation of SRRM2 at Ser1068 and impairment of RNA splicing occurred prior to the accumulation or aggregation of disease proteins.

In mass analysis, pSer1068-SRRM2 increased by approximately 1.3-fold in whole cerebral cortex tissues including non-neuronal cells (Fig. 1b). In western blot of whole cerebral cortex, pSer1068-SRRM2 increased by 4 folds (Fig. 1d). In immunohistochemistry, pSer1068-SRRM2 stain signals in neuronal cytoplasm were increased by more than 10-fold (Fig. 1c). Western blot of neurons, glia and other cells obtain from whole cerebral cortex by FACS (Fig. 1e) explained the gap between western blot and immunohistochemistry. Though the gap between mass analysis with a high threshold and western blot based on enhanced signals remains, all three experiments supported the increase of SRRM2 phosphorylation at Ser1068. The resulting decrease of the scaffold protein SRRM2 destabilized multiple splicing-related proteins including PQBP1 and SC35, impairing cellular functions that depend on these nuclear proteins. Among them, our in vivo data strongly suggested that changes in PQBP1 had a great impact on synapse and cognitive function in the pathology of AD.

PQBP1 is a causative gene for syndromic and non-syndromic X-linked ID [7–10]. Therefore, the decreased density of dendritic spines in the cerebral cortex of Synapsin-Cre *Pqbp1*-cKO mice (Fig. 4a-d) could be a pathological basis for *PQBP1*-linked ID. A similar reduction of dendritic spines in 5xFAD AD model mice and the recovery of dendritic spine density by AAV-PQBP1 suggests AD, a neurodegenerative dementia, and *PQBP1*-linked intellectual disability (ID), a developmental disease, share a common pathology. We also reveal the underlying mechanism for how molecular changes in the ID gene product lead to a reduction in the number of dendritic spines in AD. Hence, this would be the first report to show a direct molecular connection between an ID gene and AD. Our results also suggest that facilitating the function of the ID gene in adulthood could rescue the synapse pathology of AD and ameliorate cognitive symptoms.

Regarding the nuclear translocation of SRRM2, we identified TCP1α or the TRiC chaperonin complex as a critical factor in AD pathology. It is noteworthy that the TRiC complex is implicated in polyglutamine diseases [40]. The TRiC complex reduces toxicity by correcting the misfolded structures of huntigntin [40] and activates its degradation by autophagy [41]. On the other hand, inhibition of the co-chaperone Hsp90 ameliorates the symptoms of spinal-bulbar muscular atrophy [42], suggesting that the roles of the TRiC complex in neurodegeneration are complex and inconsistent. This study provides an alternative viewpoint for the involvement of the TRiC complex in neurodegeneration, according to which TRiC regulates the normal function of SRRM2, contributing to the pre-aggregation pathology triggered by non-aggregated cytoplasmic disease proteins.

A remaining question is what constitutes the upstream pathway of SRRM2 phosphorylation. We identified Erk1/2 as kinases responsible for SRRM2 phosphorylation at Ser1068. The pathway upstream of Erk1/2 activation is assumed to be extracellular Aβ in the “amyloid hypothesis”, but this is not consistent with our results. Intracellular Aβ accumulation is obviously an alternative candidate for triggering Erk1/2 activation, SRRM2 phosphorylation at Ser1068, and reductions in the levels of SRRM2/PQBP1, which is supported by our results showing co-immunostaining of Erk1/2 and other relevant factors with Aβ (Supplementary Fig. 10–13). In addition, this theory is consistent with data showing that ER stress activates Erk1/2 [43]. Interestingly, Erk1/2 are also involved in the TLR4-signaling pathway, and HMGB1, a representative DAMP molecule and a well-known ligand for TLR4, could be involved in the early stage of AD pathology [44]. Therefore, signals from intracellular ER stress and extracellular alarmins present before the formation of extracellular Aβ aggregates might merge to activate Erk1/2. However, many questions remain concerning the upstream cascade leading to SRRM2 phosphorylation, and further investigations will be required to understand “pre-aggregation” AD pathology.

## Electronic supplementary material

Integrated Sup Figures with Legends (cleared)

Supplementary Figure 1

Supplementary Figure 2

Supplementary Figure 3

Supplementary Figure 4

Supplementary Figure 5

Supplementary Figure 6

Supplementary Figure 7

Supplementary Figure 8

Supplementary Figure 9

Supplementary Figure 10

Supplementary Figure 11

Supplementary Figure 12

Supplementary Figure 13

Supplementary Figure 14

Supplementary Figure 15

Supplementary Video

Supplementary Table 1

Supplementary Table 2
