# Supplementary material for: The intellectual disability gene PQBP1 rescues Alzheimer’s disease pathology
Source: Mol Psychiatry. 2018 Oct 3;23(10):2090–110. doi: 10.1038/s41380-018-0253-8 (PMC6250680; doi:10.1038/s41380-018-0253-8)
Supplement: Supplementary file 1 — Integrated Sup Figures with Legends (cleared) [file 41380_2018_253_MOESM1_ESM.pdf]

Supplementary Information for

The intellectual disability gene PQBP1 rescues Alzheimer's disease pathology

Hikari Tanaka[1, #], Kanoh Kondo[1, #], Xigui Chen[1, #], Hidenori Homma[1, #], Kazuhiko Tagawa[1], Aurelian Kerever[2], Shigeki Aoki[2], Takashi Saito[3], Takaomi Saido[3], Kyota Fujita[1, #], and Hitoshi Okazawa[1, 4, $]

1: Department of Neuropathology, Medical Research Institute, Tokyo Medical and Dental University, 1-5-45 Yushima, Bunkyo-ku, Tokyo 113-8510, Japan
2: Department of Radiology, Juntendo University School of Medicine, 2-1-1 Hongo, Bunkyo-ku, Tokyo 113-8421, Japan
3: Laboratory for Proteolytic Neuroscience, Brain Science Institute, RIKEN, 2-1 Hirosawa, Wako, Saitama 351-0198, Japan
4: Center for Brain Integration Research, Tokyo Medical and Dental University, 1-5-45 Yushima, Bunkyo-ku, Tokyo 113-8510, Japan

#: These authors contributed equally to this work.
$: Correspondence should be addressed to H.O.
E-mail: okazawa-tky@umin.ac.jp

This PDF file includes:

Supplementary figures and legends

Supplementary Figure 1

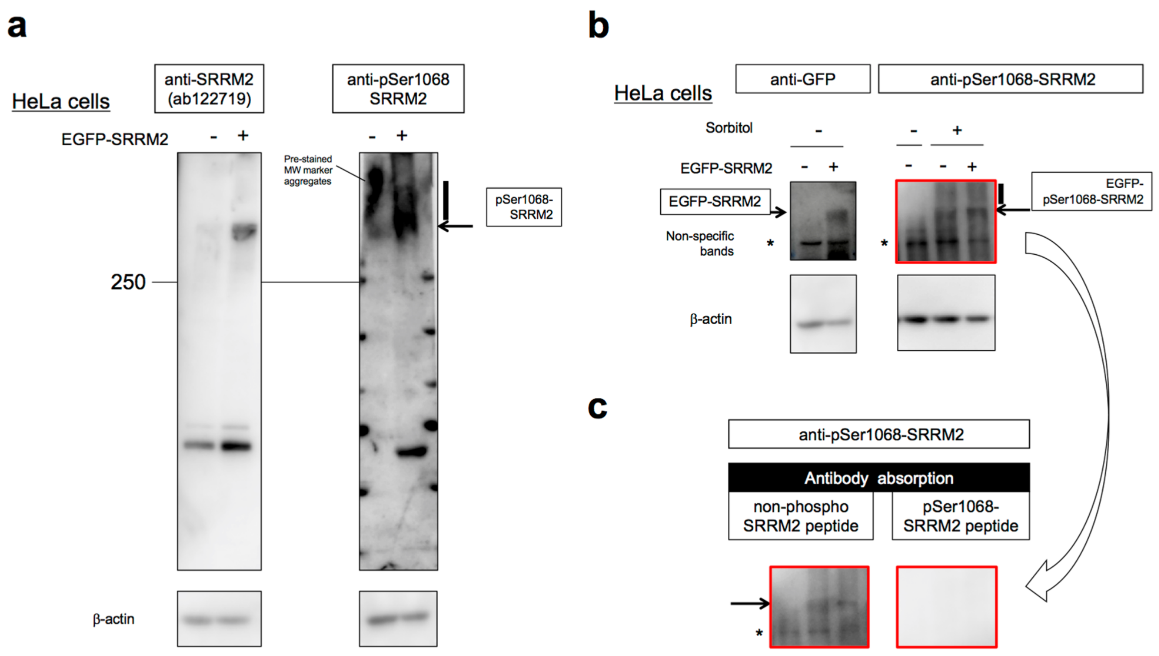

Supplementary Figure 1
Characterization of anti-pSer1068-SRRM2 antibody
a. An anti-pSer1068-SRRM2 polyclonal antibody was generated and used for western blotting of extracts of HeLa cells transiently expressing EGFP-SRRM2. A band and a smear above the MW of EGFP-SRRM2 were detected with the anti-pSer1068-SRRM2 polyclonal antibody.
b. The anti-pSer1068-SRRM2 antibody detected osmotic stress-induced pSer1068-SRRM2 in HeLa cells treated with sorbitol. Induction of pSer1068-SRRM2 in non-transfected HeLa cells was also detected with the newly generated antibody.
c. The anti-pSer1068-SRRM2 polyclonal antibody was incubated with non-phosphorylated SRRM2 peptide or the SRRM2 peptide phosphorylated at Ser1068 used for the generation of the antibody. The bands disappeared after incubation with the phosphorylated SRRM2 peptide but not with the non-phosphorylated peptide of the same sequence.

Supplementary figure 2

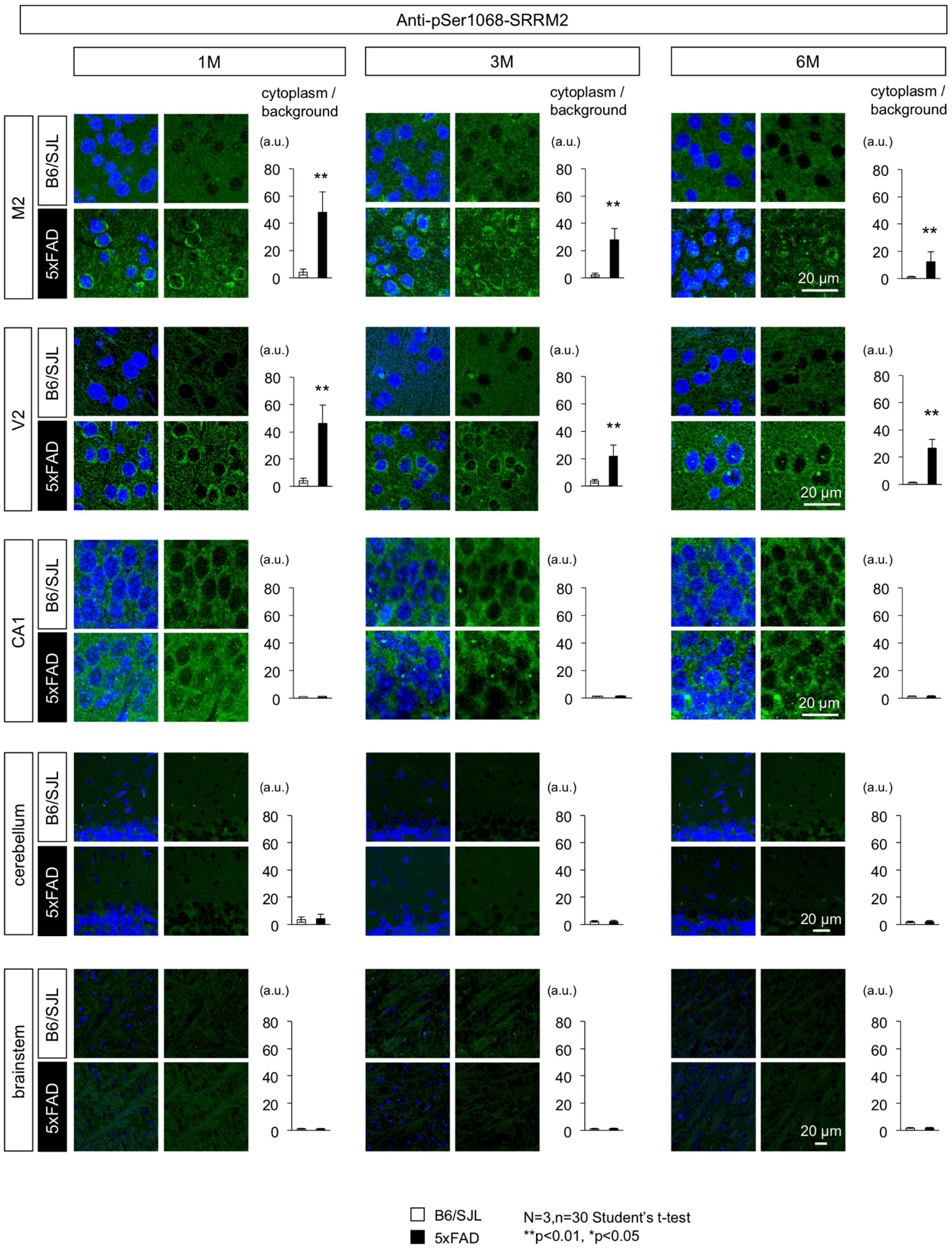

Supplementary Figure 2

SRRM2 phosphorylation at pSer1068 was increased in specific brain regions

Immunohistochemistry of 5xFAD and B6/SJL mice at 1, 3, and 6 months of age revealed an increase of pSer1068-SRRM2 in neurons. Supplementary data to Figure 1c are shown. Cytoplasmic staining of neurons was detected in secondary motor cortex (M2), secondary visual cortex (V2), and the other cortex areas. Cytoplasmic staining of neurons at field CA1 in the hippocampus (CA1) did not change considerably at 1 and 3 months, although some cytoplasmic granules of pSer1068-SRRM2 were detected in 5xFAD mice at 6 months. No definite stains of pSer1068-SRRM2 were detected in the cerebellum and brain stem. Quantitative analyses of intensities are shown in right graphs at each time point and in each area. Signal intensities were determined in six cytoplasmic areas of a single cell, and the mean was adjusted to the background intensity. The corrected mean values from 30 cells in each brain area were used to calculate the representative value of a mouse. Statistical analysis was performed with the values of three mice in each area at each time point by Student's t-test.

Supplementary Figure 3

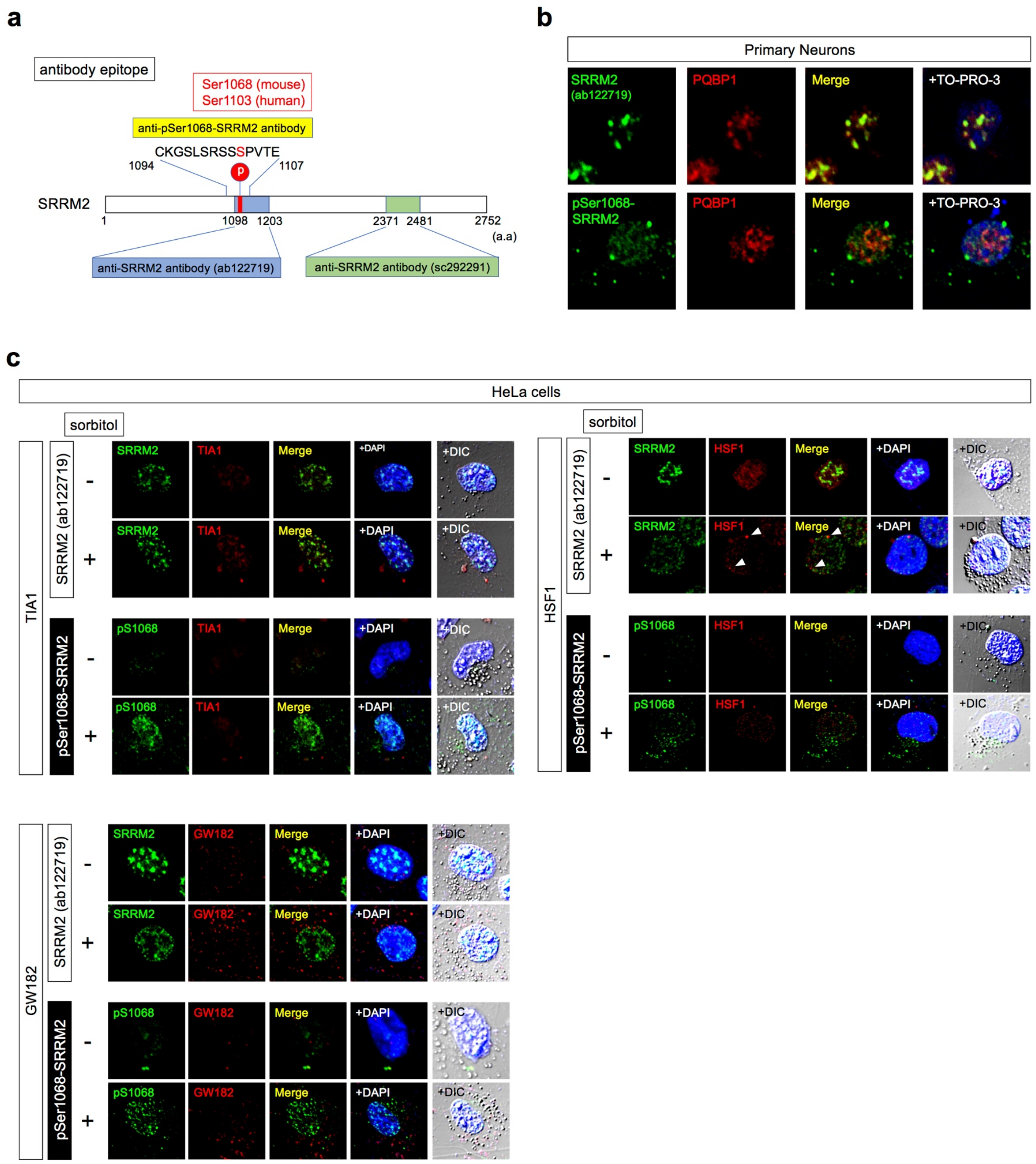

Supplementary Figure 3

Phosphorylation at Ser1068 shifts SRRM2 to the cytoplasm

a. Antigen regions of two anti-SRRM2 antibodies and anti-pSer1068-SRRM2 antibody.

b. Anti-pSer1068-SRRM2 antibody stained cytoplasmic granules of pSer1068-SRRM2, while anti-SRRM2 antibody (ab122719) stained nuclear speckles of non-phosphorylated SRRM2.

c. High osmolarity increased pSer1068-SRRM2 in cytoplasm. The pSer1068-SRRM2 did not merge with cytoplasmic stress granule (TIA), P-body (GW182) or nuclear stress granule (HSF1).

Supplementary Figure 4

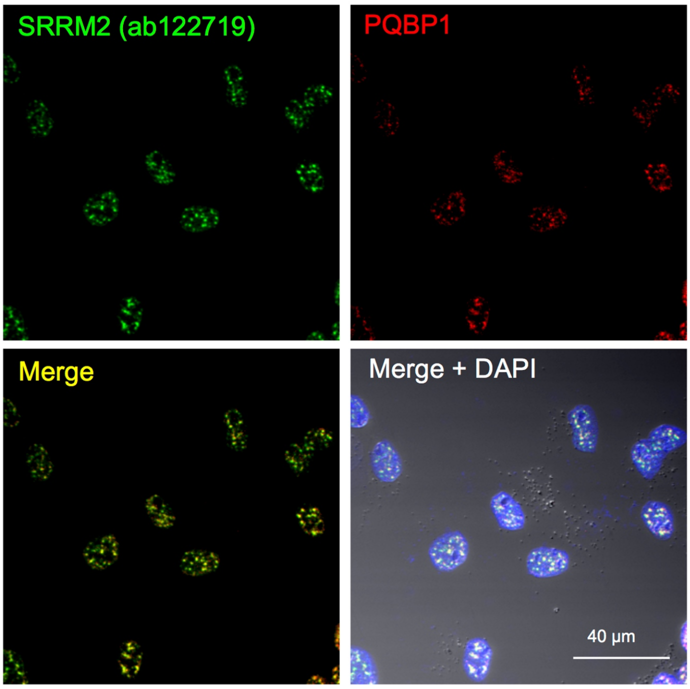

Supplementary Figure 4
Co-localization of SRRM2 and PQBP1 at nuclear speckles
Immunocytochemistry of untreated HeLa cells with anti-SRRM2 and anti-PQBP1 antibodies. SRRM2 and PQBP1 co-localized at small speckles in the nucleus.

Supplementary Figure 5

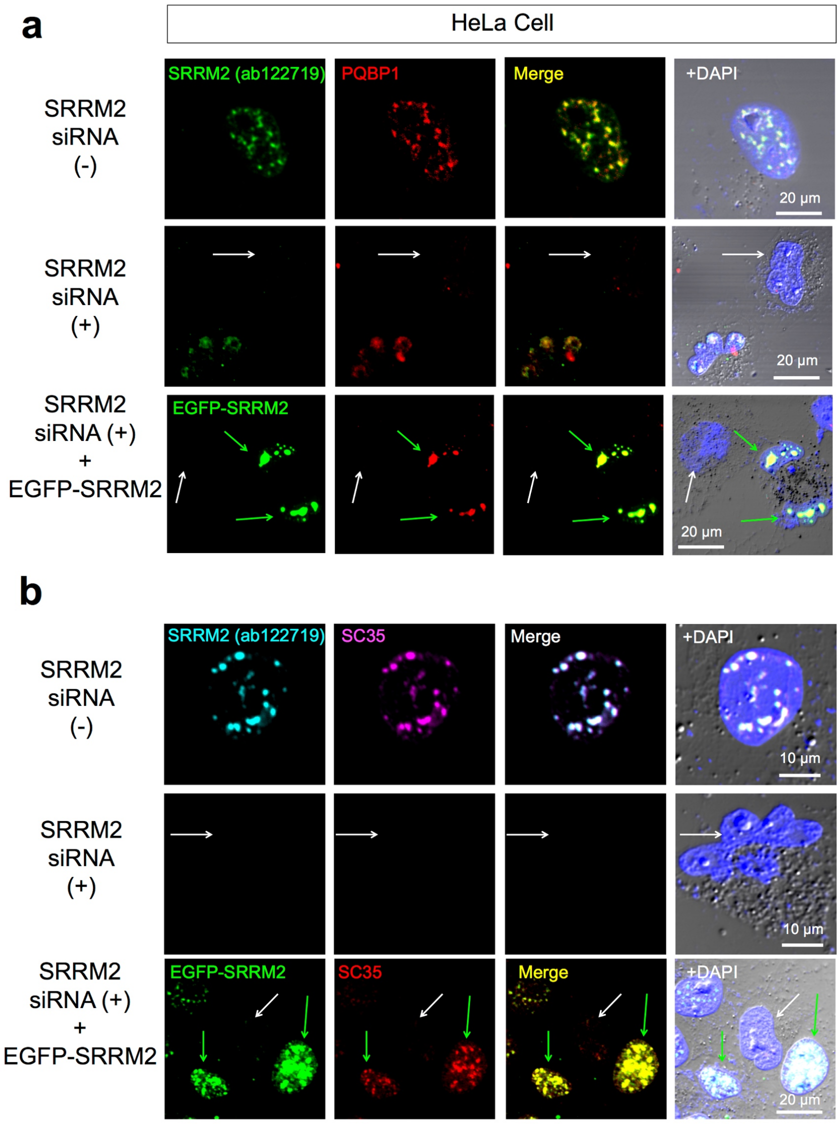

Supplementary Figure 5
Knockdown of SRRM2 reduces PQBP1 and SC35
a.b. SiRNA-mediated SRRM2 knockdown also reduced PQBP1 **(a)** and SC35 **(b)** in HeLa cells (white arrow), whereas recovery of SRRM2 protein levels by EGFP-SRRM2 transient expression rescued PQBP1 and SC35 (green arrow).

Supplementary Figure 6

a

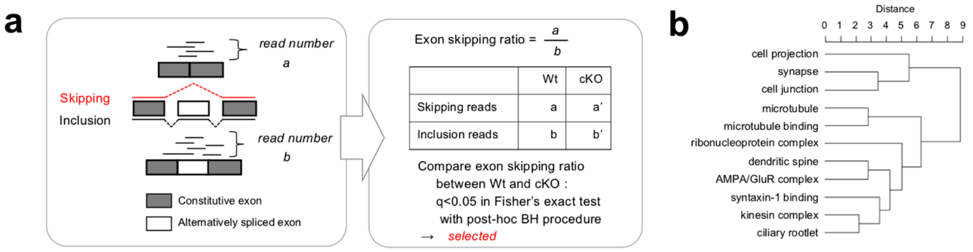

c

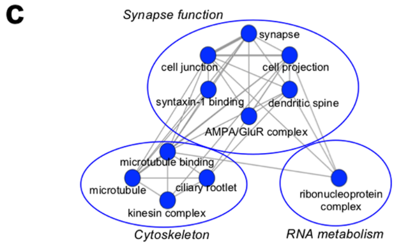

d

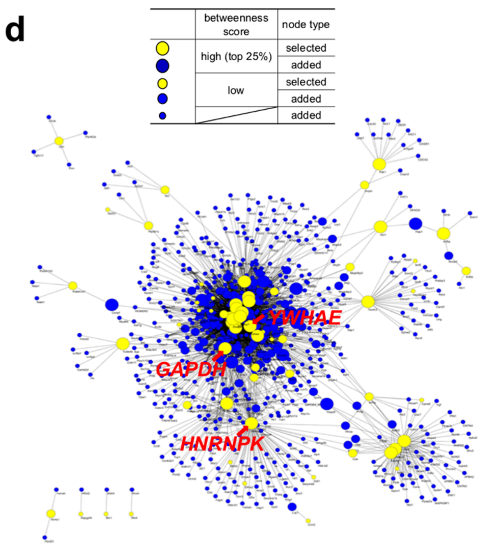

e

Gene symbol	Exon number	Betweenness	Exon skipping ratio	Separation score	p-value	q-value
Gapdh	2	0.2199	17.0758	4.0939	4.E-10	5.E-07
Ywhae	3	0.2150	0.1939	-2.3668	2.E-17	4.E-14
Hnrnpk	2	0.1537	3.1249	1.6438	8.E-06	0.0037
Dlg4	3	0.1235	1.8236	0.8668	9.E-06	0.0043
App	10	0.1072	0.7154	-0.4831	2.E-10	2.E-07
App	11	0.1072	0.0105	-6.5739	3.E-80	6.E-76
App	12	0.1072	0.0068	-7.1966	2.E-83	5.E-79
Camk2a	9	0.0912	5.3858	2.4292	1.E-72	2.E-68
Camk2a	8	0.0912	1.8784	0.9095	5.E-22	1.E-18
Ptk2b	22	0.0871	1.5680	0.6490	0.0001	0.0400
Mbp	6	0.0673	1.2019	0.2653	1.E-06	0.0006
Dlg2	5	0.0643	6.3766	2.6728	5.E-05	0.0177
Tenm3	20	0.0616	0.0980	-3.3505	9.E-05	0.0311
Hnrnpa2b1	6	0.0491	0.6626	-0.5939	1.E-09	1.E-06
Nsf	2	0.0410	0.4290	-1.2210	1.E-09	1.E-06
Nsf	3	0.0410	0.0680	-3.8778	6.E-24	2.E-20
Dnm1	2	0.0332	1.9010	0.9267	5.E-26	2.E-22
Dnm1	12	0.0332	1.5373	0.6204	9.E-17	2.E-13
Dnm1	11	0.0332	1.4189	0.5048	8.E-08	6.E-05
Dnm1	10	0.0332	0.6889	-0.5376	5.E-15	1.E-11
Dnm1	13	0.0332	0.2584	-1.9524	3.E-75	6.E-71
Pak1	10	0.0269	2.0665	1.0472	2.E-07	0.0001
Klc1	6	0.0241	0.6213	-0.6866	2.E-08	2.E-05
Cttn	12	0.0224	0.0654	-3.9343	4.E-09	3.E-06
Syt1	7	0.0213	39.5533	5.3057	1.E-37	9.E-34
Syt1	8	0.0213	31.2930	4.9678	6.E-29	3.E-25
Snap25	5	0.0211	1.1595	0.2135	1.E-05	0.0054
Tubb4b	3	0.0135	19.2883	4.2697	1.E-05	0.0065
Kif5a	19	0.0135	0.1392	-2.8443	5.E-38	4.E-34
Glul	2	0.0105	32.8442	5.0376	7.E-17	2.E-13
Glul	3	0.0105	31.4167	4.9735	3.E-16	6.E-13
Gabbr1	8	0.0093	2.9780	1.5744	6.E-05	0.0219
Gabbr1	7	0.0093	2.4250	1.2780	6.E-05	0.0214
Gabbr1	15	0.0093	2.1941	1.1336	8.E-05	0.0270
Sparcl1	2	0.0093	0.8742	-0.1940	0.0001	0.0400
Stxbp1	19	0.0090	1.7071	0.7715	4.E-10	4.E-07

App: Amyloid beta A4 protein, Camk2a: Calcium/calmodulin-dependent protein kinase type II subunit alpha, Cttn: Src substrate cortactin, Dlg2: Disks large homolog 2, Dlg4: Disks large homolog 4, Dnm1: Dynamin-1, Gabbr1: Gamma-aminobutyric acid type B receptor subunit 1, Gapdh: Glyceraldehyde-3-phosphate dehydrogenase, Glul: Glutamine synthetase, Hnrnpa2b1: Heterogeneous nuclear ribonucleoproteins A2/B1, Hnrnpk: Heterogeneous nuclear ribonucleoprotein K, Kif5a: Kinesin heavy chain isoform 5A
Klc1: Kinesin light chain 1, Mbp: Myelin basic protein, Nsf: Vesicle-fusing ATPase, Pak1: Serine/threonine-protein kinase PAK 1, Ptk2b: Protein-tyrosine kinase 2-beta, Snap25: Synaptosomal-associated protein 25, Sparcl1: SPARC-like protein 1, Stxbp1: Syntaxin-binding protein 1, Syt1: Synaptotagmin-1, Tenm3: Teneurin-3, Tubb4b: Tubulin beta-4B chain, Ywhae: 14-3-3 protein epsilon

Supplementary Figure 6
PQBP1 deficiency impacts synapses by changing RNA splicing

a. The concept of exon skipping and calculation of the skipping ratio. The exon skipping ratios of whole exons from all genes were compared between wild-type male and Synapsin-Cre PQBP1-cKO male mice, and genes containing more than one exon that changed significantly in Fisher's exact test were selected for further analyses.

b. Gene ontology (GO) enrichment analysis of selected genes in **a**.

c. Network analysis of GO-enriched groups indicated neuron-specific PQBP1-deficiency affects mainly synapse function, cytoskeleton and RNA metabolism.

d. A functional network of genes showing pathologically altered splicing under conditions of PQBP1 deficiency in mature neurons (Synapsin-Cre PQBP1-cKO) was generated based on the integrated protein-protein interaction database (http://genomenetwork.nig.ac.jp/index_e.html). The high quality figure is posted at http://suppl.atgc.info/021/.

e. Core genes were selected based on the betweenness score in the pathological network under conditions of PQBP1 deficiency in mature neurons. The top three core genes with the highest betweenness scores were GAPDH, Ywhae (14-3-3 ε), and hnRNPK. APP was ranked at the 5th position.

Supplementary Figure 7

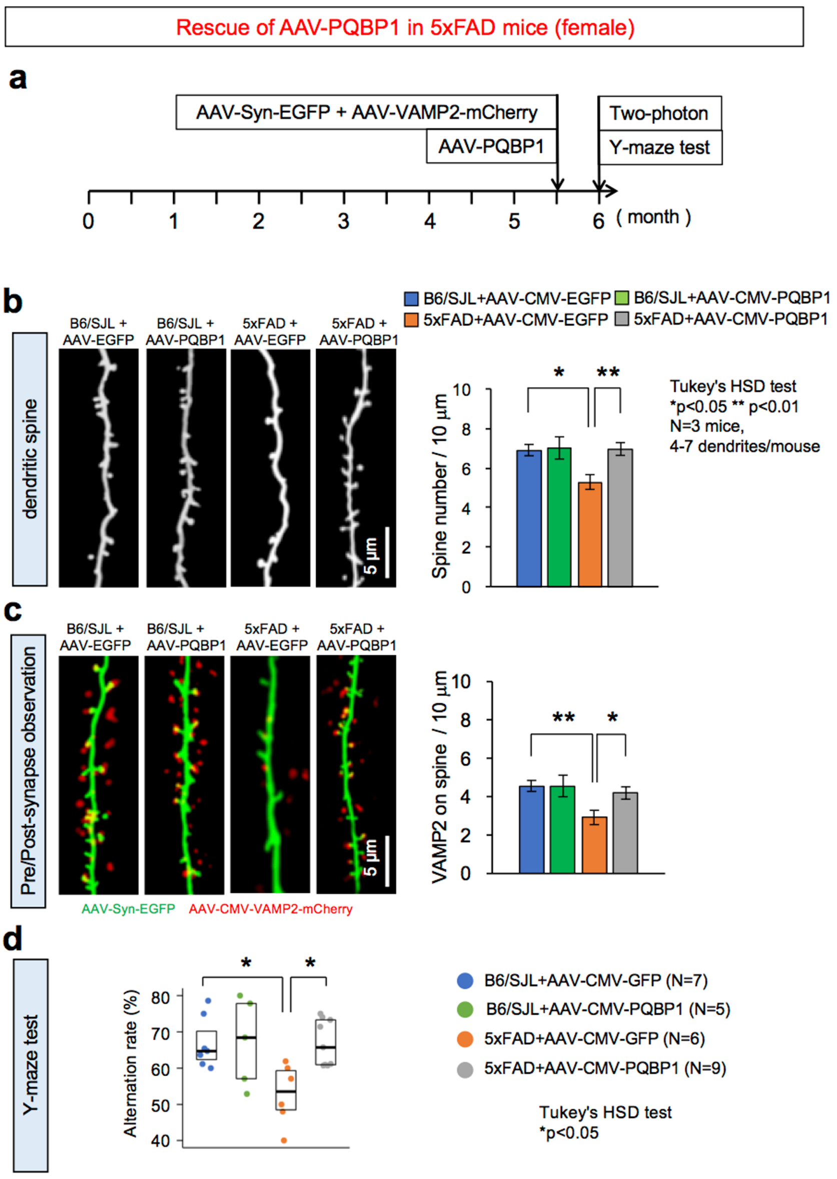

Supplementary Figure 7

PQBP1 rescues synapse and cognitive function of female 5xFAD mice

a. AAV-PQBP1 mediated rescue of synapse pathology in 5xFAD female mice. 5xFAD female mice received a single injection of AAV-PQBP1 into RSD at 5.5 months and were evaluated 2 weeks later at 6 months of age. The protocol was exactly similar to that for male 5xFAD mice.

b. Two-photon microscopic images of dendritic spines in the first layer of RSD in 5xFAD or B6/SJL female mice after injection of AAV-EGFP or AAV-PQBP1. The right graph shows the quantitative analysis of spine number.

c. Two-photon microscopic images of contact of axon terminals and dendritic spines in the first layer of RSD in 5xFAD female mice after injection of AAV-Vamp-Cherry with AAV-EGFP or AAV-PQBP1. The right graph shows the quantitative analysis of the axon terminals merged on the spine.

d. Alteration ratios in the Y-maze test of 5xFAD female mice after injection of AAV-EGFP or AAV-PQBP1 are shown.

Supplementary Figure 8

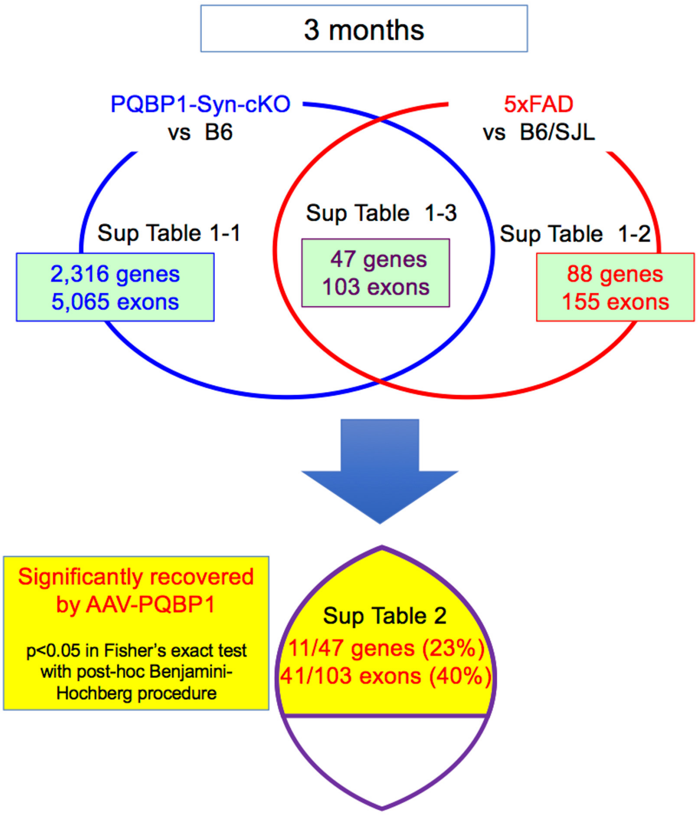

Supplementary Figure 8
AAV-PQBP1 rescues RNA splicing of synapse-related genes in 5xFAD mice
We compared genes changed in splicing (p <0.05 in Fisher's exact test, q-value is also sown) between PQBP1-Syn-Cre mice and 5xFAD mice. 47 genes and their 103 exons were commonly changed in two mouse models. The lower Ben graph shows recovery of the abnormal RNA splicing by AAV-PQBP1. 41 in 103 exons and 11 in 47 genes were recovered with a statistical significance. Corresponding Supplementary Table and Sheet Numbers are indicated.

Supplementary Figure 9

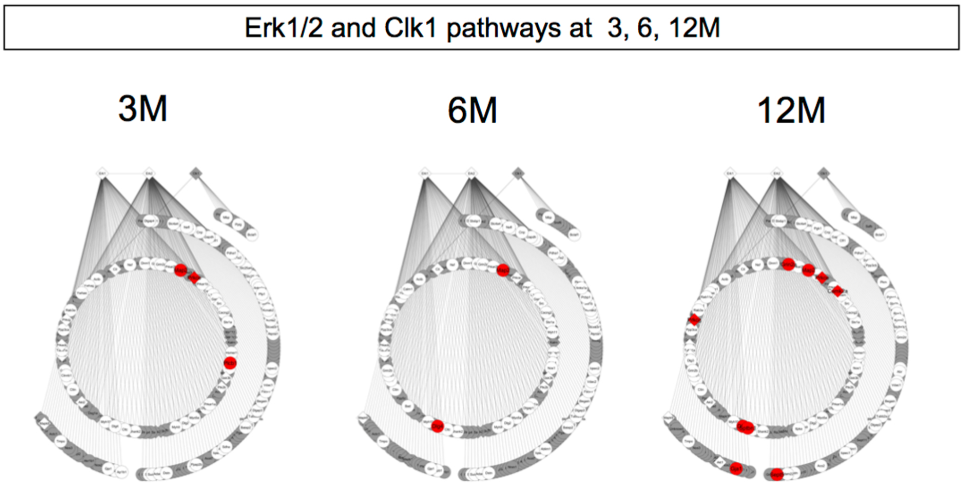

	3M		6M		12M	
	Erk1/2 downstream	Erk1/2 independent	Erk1/2 downstream	Erk1/2 independent	Erk1/2 downstream	Erk1/2 independent
Phosphoproteins	3	13	2	15	9	49
Detected proteins	100	1145	94	964	95	960
Ratio	0.030	0.011	0.021	0.016	0.095	0.051
p-value	0.131		0.658		0.094	

	3M		6M		12M	
	Clk1 downstream	Clk1 independent	Clk1 downstream	Clk1 independent	Clk1 downstream	Clk1 independent
Phosphoproteins	0	16	0	17	0	58
Detected proteins	4	1241	2	1056	2	1053
Ratio	0	0.013	0	0.016	0	0.055
p-value	1		1		1	

Supplementary Figure 9
The Erk1 pathway was not significantly activated at later AD stages
Pathway analysis of downstream target proteins of Erk1/2 and Clk1. Proteins shown in red indicate increased phosphorylation at 3, 6, and 12 month of age in whole cerebral cortex tissues of 5xFAD. The high quality figures are posted at http://suppl.atgc.info/021/.

Supplementary Figure 10

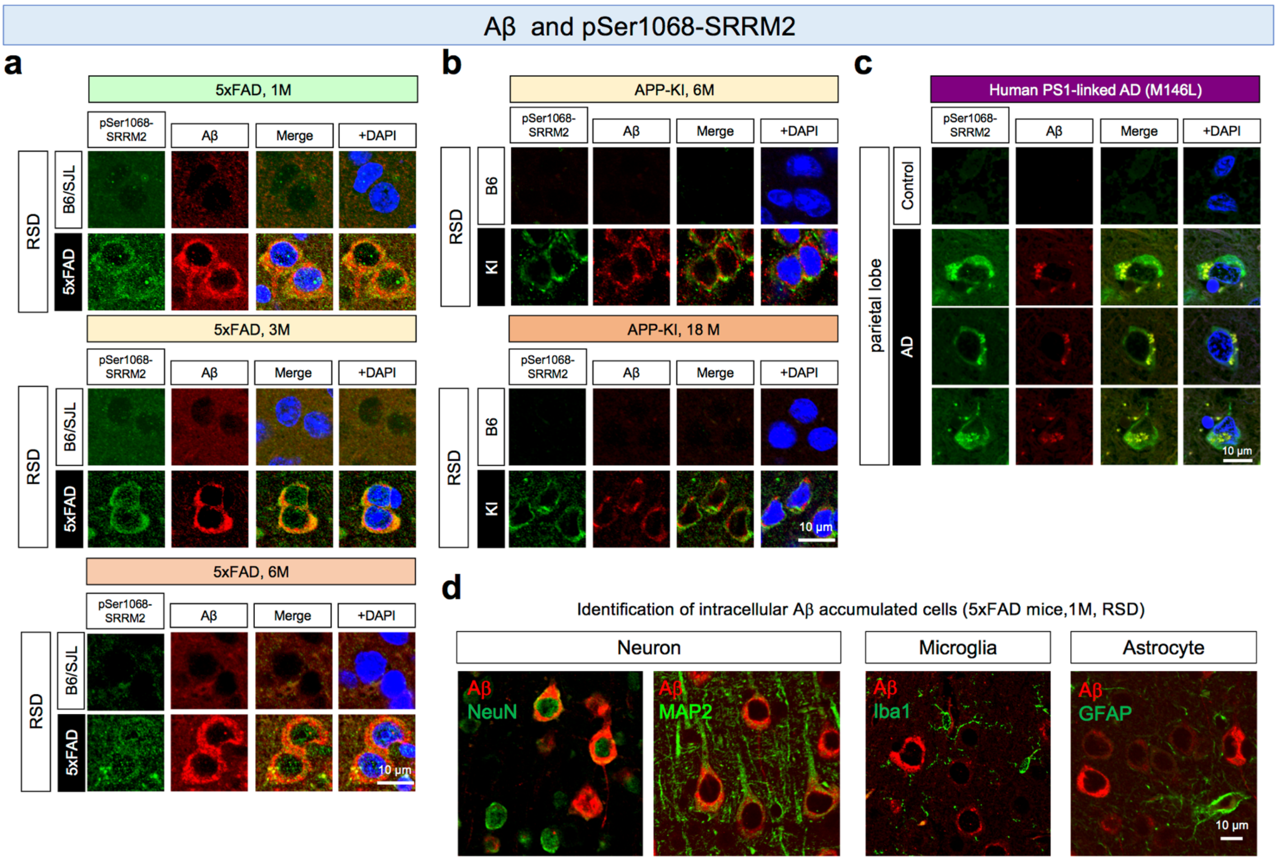

Supplementary Figure 10

Aβ and pSer1068-SRRM2 in 5xFAD mice, APP-KI mice, and human AD brains

a. Co-staining of pSer1068-SRRM2 and intracellular Aβ was detected widely in cortical neurons of 5xFAD mice at 1–6 months of age.

b. The co-localization of pSer1068-SRRM2 and intracellular Aβ was also confirmed in neurons of the cerebral cortex in APP-KI mice at 6 and 18 months.

c. The similar co-localization was confirmed in cortical neurons of postmortem PS1-mutated human AD brains. In **a-c**, the 6E10 antibody was used to stain intracellular Aβ, which is sometimes less sensitive than 82E1, to exclude false-positive reactivity.

d. Co-staining with MAP2 or NeuN confirmed that intracellular Aβ-positive cells were neurons. Microglia and astrocyte markers did not merge with intracellular Aβ-positive cells.

Supplementary Figure 11

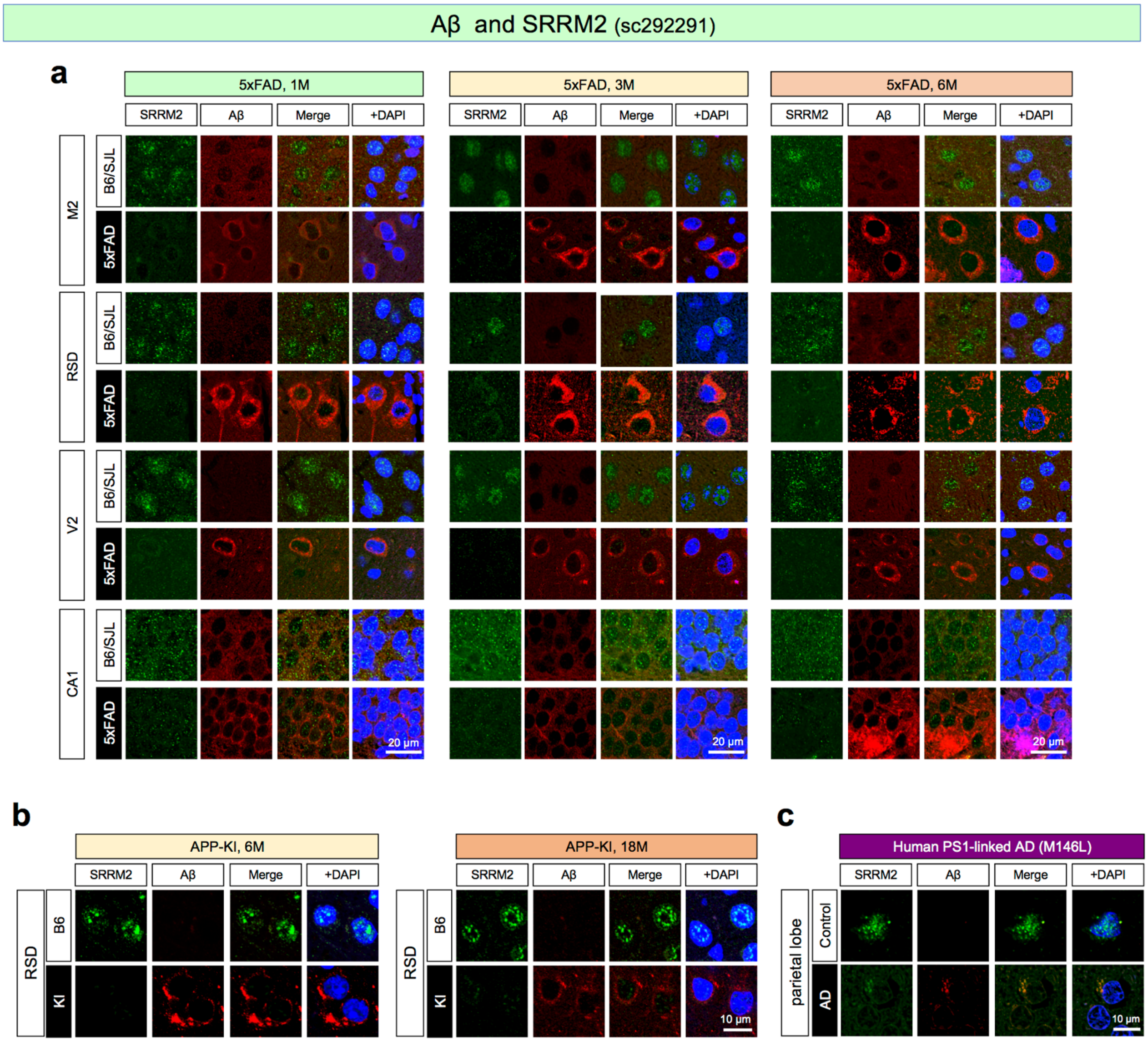

Supplementary Figure 11
Aβ and SRRM2 in 5xFAD mice, APP-KI mice, and human AD brains
a-c. Co-staining of Aβ and SRRM2 revealed that SRRM2 was generally lower in cortical neurons with intracellular Aβ accumulation in various regions in the brain of 5xFAD mice at 1–6 months of age **(a)**. M2: secondary motor cortex, RSD: retrosplenial dysgranular cortex, V2: secondary visual cortex, CA1: field CA1 hippocampus. The decrease of SRRM2 in cortical neurons with intracellular Aβ was confirmed in the cortical neurons of APP-KI mice at 6 and 18 months **(b)** as well as in cortical neurons of postmortem PS1-mutated human AD brains **(c)**.

Supplementary Figure 12

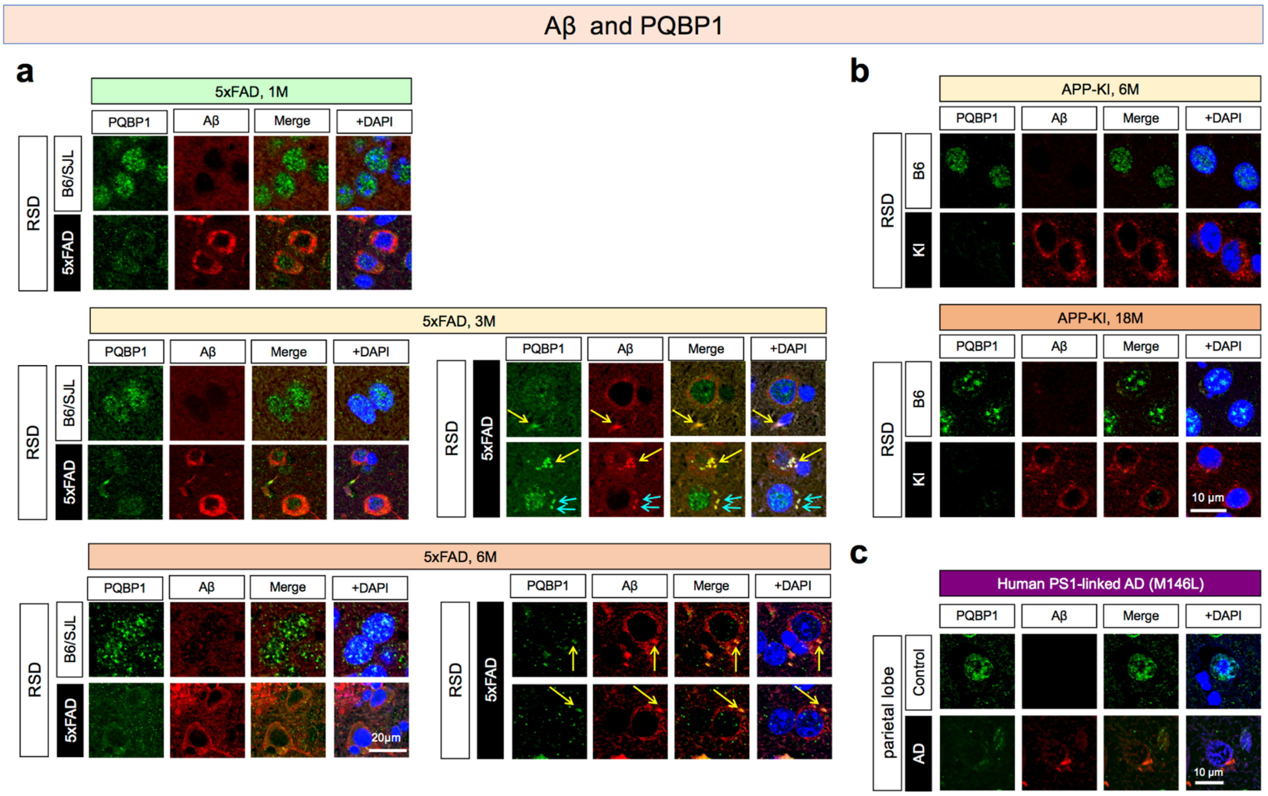

Supplementary Figure 12

Aβ and PQBP1 in 5xFAD mice, APP-KI mice, and human AD brains

a. Co-staining of Aβ and PQBP1 revealed that the PQBP1 protein was lower in cortical neurons of 5xFAD mice with diffuse intracellular Aβ than in cortical neurons of sibling B6/SJL mice (left panels). Starting at 3 months of age, some cortical neurons of 5xFAD mice showed aggregate-like cytoplasmic foci of Aβ (right panels), which partly coincided with PQBP1 downregulation (yellow arrow), whereas in other sections, the PQBP1 protein level remained equivalent to the normal level. PQBP1 co-localized with aggregate-like Aβ cytoplasmic foci.

b. c. The downregulation of PQBP1 in cortical neurons with intracellular Aβ was confirmed in the RSD of APP-KI mice at 6 and 18 months **(b)** as well as in the parietal lobe of postmortem PS1-mutated human AD brains **(c)**.

Supplementary Figure 13

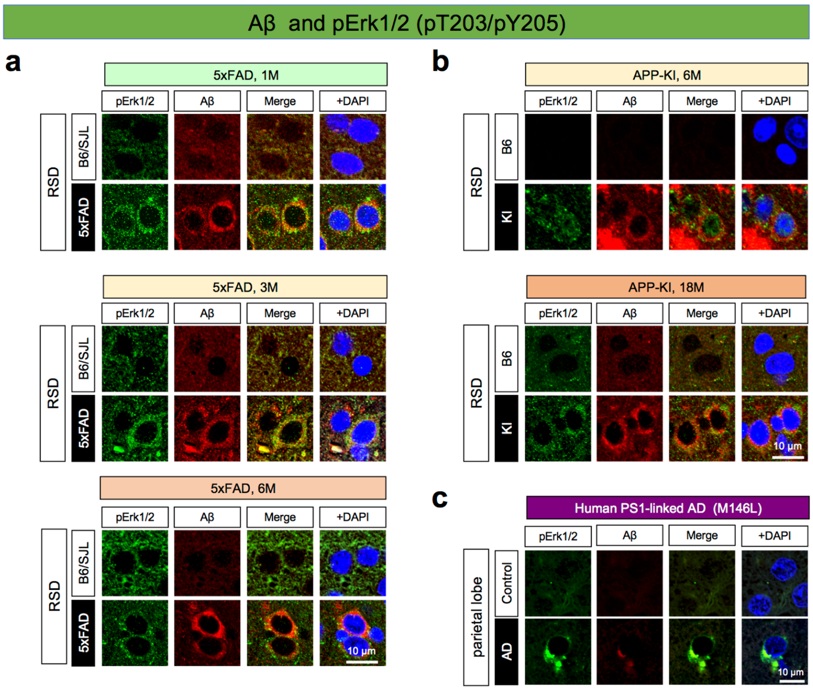

Supplementary Figure 13

Aβ and activated Erk1 in 5xFAD mice, APP-KI mice and human AD brains

a-c. Co-staining of Aβ and the active forms of Erk1/2 (pT203/pY205) revealed that Erk1/2 were activated in cortical neurons with intracellular Aβ of 5xFAD mice **(a)**, APP-KI mice **(b),** and human AD brains with the PS1 Met146Leu mutation **(c)**.

Supplementary Figure 14

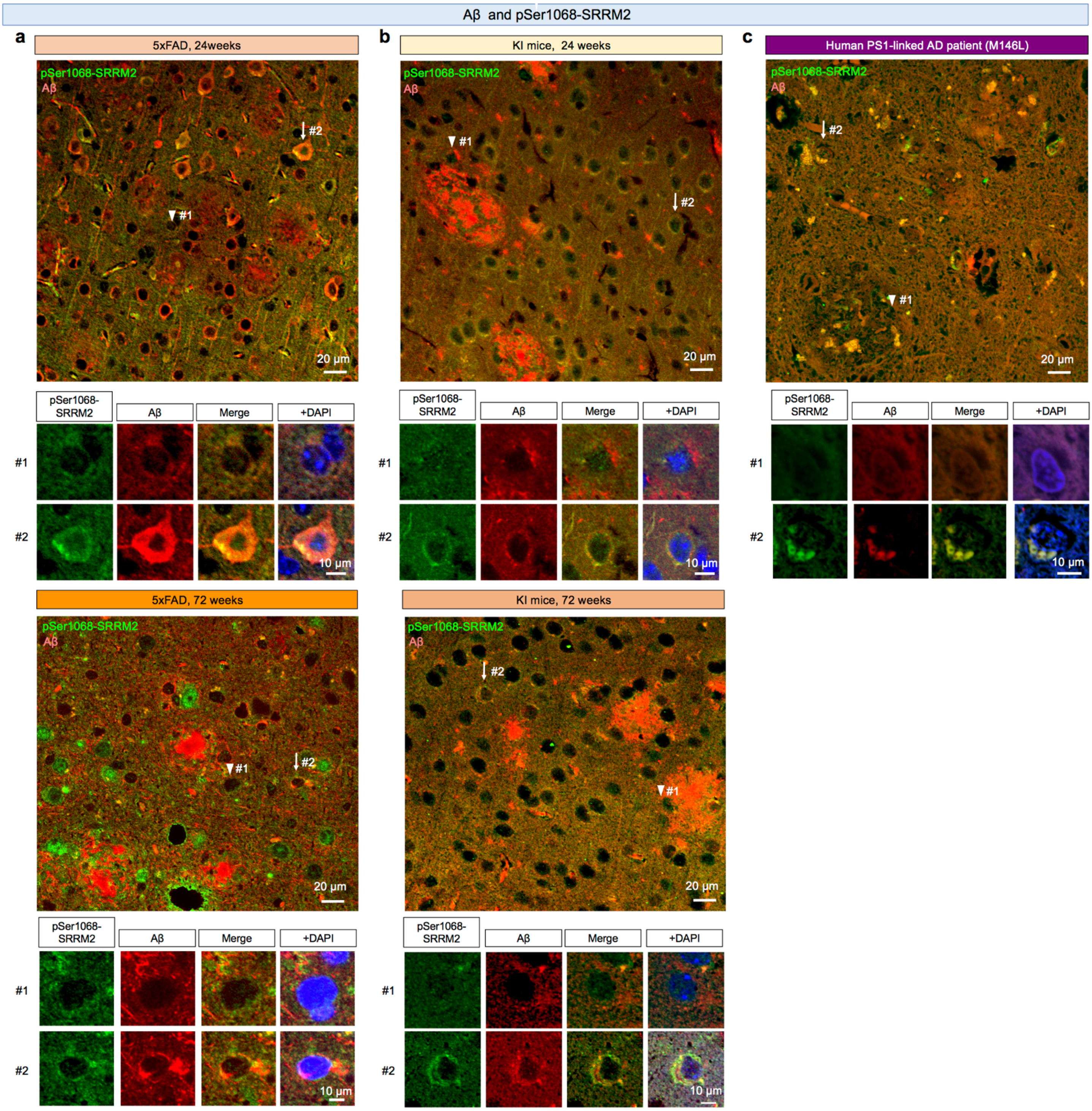

Supplementary Figure 14

Aβ and pSer1068-SRRM2 in 5xFAD mice, APP-KI mice and human AD patients

a-c. Low-power-magnification images of 5xFAD mice **(a)**, APP-KI mice **(b)** or PS1-linked AD patients with the PS1 Met146Leu mutation **(c)** did not support a direct relationship between the distance from extracellular Aβ plaques and phosphorylation of SRRM2, since neurons distant from the plaques (#2) also showed high pSer46-SRRM2 signals equivalent to neurons surrounding the plaques (#1). Most of the neurons with high pSer46-SRRM2 signals were co-stained with intracellular Aβ.

Supplementary Figure 15

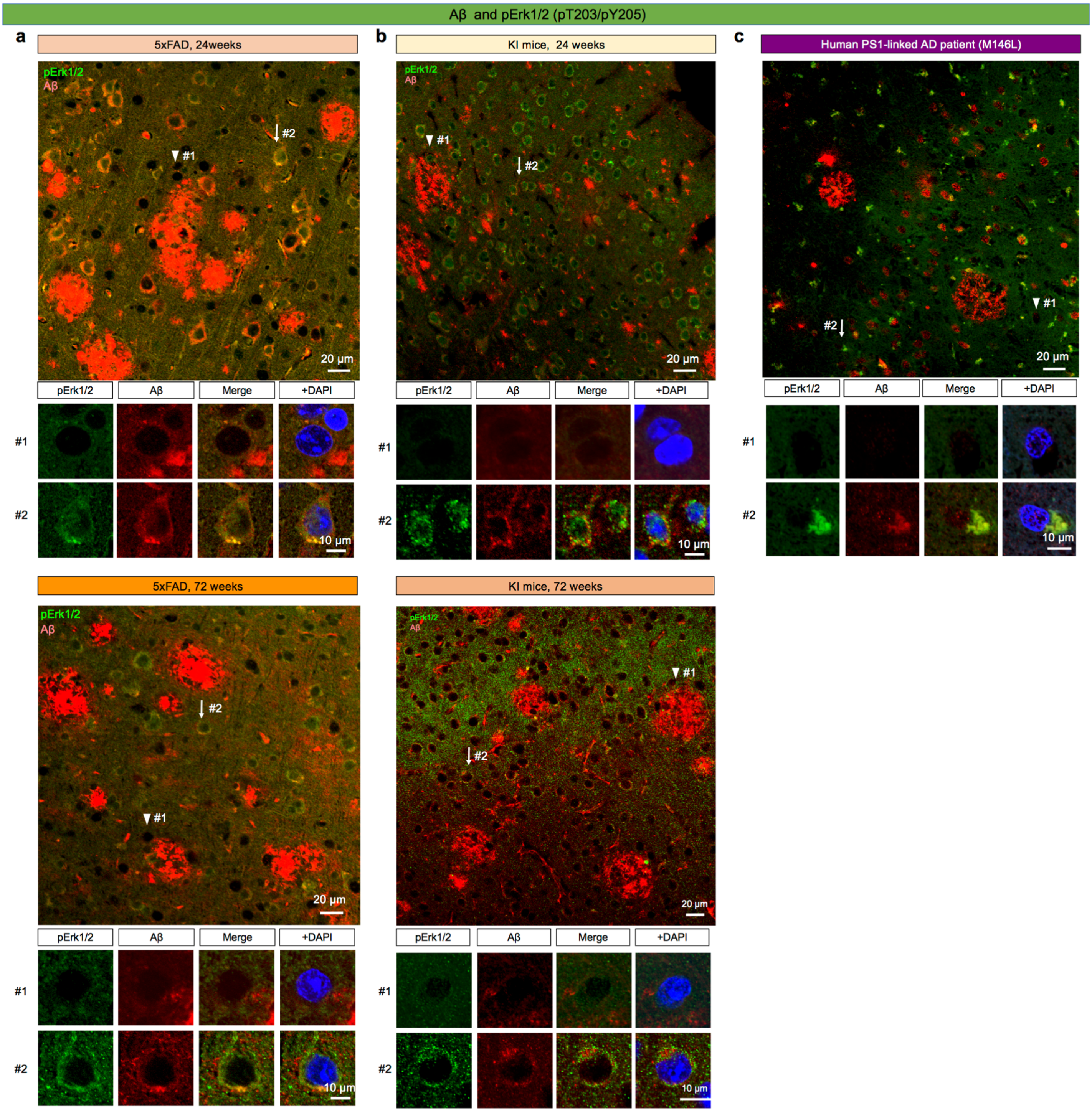

Supplementary Figure 15

Aβ and pErk1/2 in 5xFAD mice, APP-KI mice and human AD patients

a. b. Low-power-magnification images of 5xFAD mice **(a)**, APP-KI mice **(b)** or PS1-linked AD patients with the PS1 Met146Leu mutation **(c)** did not support a direct relationship between the distance from extracellular Aβ plaques and activation of Erk1/2, since neurons distant from the plaques (#2) also showed high Erk1/2 signals equivalent to neurons surrounding the plaques (#1). Most of the neurons with high Erk1/2 signals were co-stained with intracellular Aβ.

Supplementary Video

Extracellular Aβ aggregates are not directly relevant to SRRM2 phosphorylation

3D confocal microscopic image of a transparent brain of a 5xFAD mouse is shown. Extracellular Aβ aggregates did not largely influence the intensity of pSer1068-SRRM2 staining in surrounding neurons in comparison to distant neurons.
